# The new ichthyosauriform *Chaohusaurus brevifemoralis* (Reptilia, Ichthyosauromorpha) from Majiashan, Chaohu, Anhui Province, China

**DOI:** 10.7717/peerj.7561

**Published:** 2019-09-09

**Authors:** Jian-dong Huang, Ryosuke Motani, Da-yong Jiang, Andrea Tintori, Olivier Rieppel, Min Zhou, Xin-Xin Ren, Rong Zhang

**Affiliations:** 1 Department of Research, Anhui Geological Museum, Hefei, Anhui, People’s Republic of China; 2 Department of Earth and Planetary Sciences, University of California, Davis, CA, USA; 3 Department of Geology, Peking University, Beijing, People’s Republic of China; 4 Dipartimento di Scienze della Terra, Università degli Studi di Milano, Milano, Italia; 5 Center of Integrative Research, The Field Museum, Chicago, IL, USA

**Keywords:** Early Triassic, Ichthyosauromorpha, *Chaohusaurus brevifemoralis*, Majiashan, Chaohu, Anhui Province

## Abstract

A new species of ichthyosauriform is recognized based on 20 specimens, including nearly complete skeletons, and named *Chaohusaurus brevifemoralis*. A part of the specimens was previously identified as *Chaohusaurus chaoxianensis* and is herein reassigned to the new species. The new species differs from existing species of *Chaohusaurus* in a suite of features, such as the bifurcation of the caudal peak neural spine and a short femur relative to trunk length. The specimens include both complete and partially disarticulated skulls, allowing rigorous scrutiny of cranial sutures. For example, the squamosal does not participate in the margin of the upper temporal fenestra despite previous interpretations. Also, the frontal unequivocally forms a part of the anterior margin of the upper temporal fenestra, forming the most medial part of the anterior terrace. The skull of the holotype largely retains three-dimensionality with the scleral rings approximately in situ, revealing that the eyeball was uncovered in two different directions, that is, laterally and slightly dorsally through the main part of the orbit, and dorsally through the medial extension of the orbit into the skull roof. This skull construction is likely a basal feature of Ichthyosauromorpha. Phylogenetic analyses place the new species as a sister taxon of *Chaohusaurus chaoxianensis*.

## Introduction

The marine reptile clade Ichthyosauromorpha comprises two groups, namely Ichthyosauriformes that lasted for about 160 million years and spread worldwide (McGowan, 1991; Motani et al., 2015a) and Hupehsuchia, which is a small group of heavily-built reptiles known only from the Spathian (Lower Triassic) of Hubei Province, China (Young, 1972; Chen et al., 2014a). The two groups faced contrasting fates at the end of the Early Triassic but they were almost equally diverse in the Spathian, when they were relatively new to the history of life (Motani et al., 2017). Early ichthyosauriforms from that time period had been known since 1929 based on *Grippia longirostris* from Spitsbergen (Wiman, 1929, 1933), and three additional species were reported within the same century, namely *Chaohusaurus geishanensis* from China (Young & Dong, 1972), *Utatsusaurus hataii* from Japan (Shikama, Kamei & Murata, 1978), and *Parvinatator wapitiensis* from Canada (Nicholls & Brinkman, 1995). However, none of them was represented by a complete skeleton and it was not until complete body fossils were reported that we learned the bauplan of these animals—at least *Chaohusaurus* and *Utatsusaurus* appeared like lizards with flippers (Motani, You & McGowan, 1996; Motani, Minoura & Ando, 1998), unlike later ichthyosaurs that were fish-shaped. Three more species have been recognized since, namely *Gulosaurus helmi* from Canada (Cuthbertson, Russell & Anderson, 2013a), *Cartorhynchus lenticarpus* from China (Motani et al., 2015a), and *Sclerocormus breviceps* from China (Jiang et al., 2016). *Thaisaurus chonglakmanii* from Thailand (Mazin et al., 1991) may also belong to the list but further study is needed to establish the exact stratigraphy.

*Chaohusaurus* is by far the best-known genus of the Spathian ichthyosauriforms, being represented by dozens of skeletons, and more than 10 scientific papers focusing on the genus have been published (Young & Dong, 1972; Chen, 1985; Motani, You & McGowan, 1996; Motani & You, 1998a, 1998b; Maisch, 2001; Chen et al., 2013; Motani et al., 2015b, 2015c, 2018; Zhou et al., 2017). Three species are currently recognized in the genus. The type species *Chaohusaurus geishanensis* and the second species *Chaohusaurus chaoxianensis* are known from the Chaohu fauna in Anhui Province, China. The third species, *Chaohusaurus zhangjiawanensis*, is from the Nanzhang-Yuan’an fauna in Hubei Province. Of the three species, *Chaohusaurus chaoxianensis* is the most abundant, whereas the other two are known from only a few specimens each. Most of the specimens of *Chaohusaurus chaoxianensis* were collected from former limestone quarries in the Majiashan area, located north of the eastern outlet of Chaohu Lake, Anhui Province, China. Multi-year excavations that started in 2010, by a joint team from the Peking University, University of California, Davis, University of Milan, and Anhui Geological Museum, unearthed about 60 marine vertebrate specimens from the area. About 40 of the specimens were tentatively identified as *Chaohusaurus chaoxianensis* based on the presence of poorly ossified carpals or tarsals, which is among the features that distinguish the species from *Chaohusaurus geishanensis* (Motani et al., 2015c).

**Figure 1:**
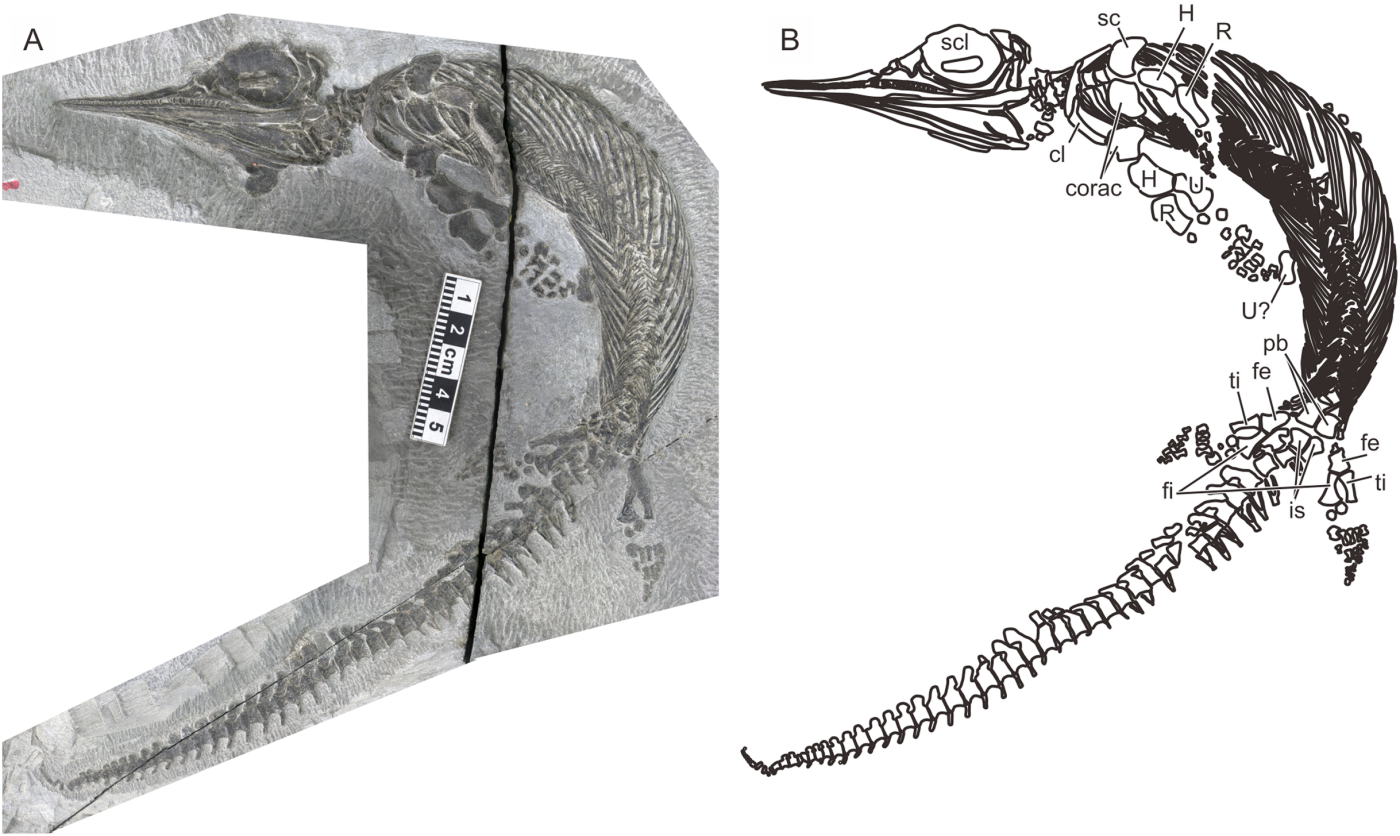
The most complete specimen of *Chaohusaurus chaoxianensis* (AGB6256). (A) Photograph. (B) Approximate bone map. See the section “Osteological abbreviations” for abbreviations. Scale bar is five cm in total.

**Figure 2:**
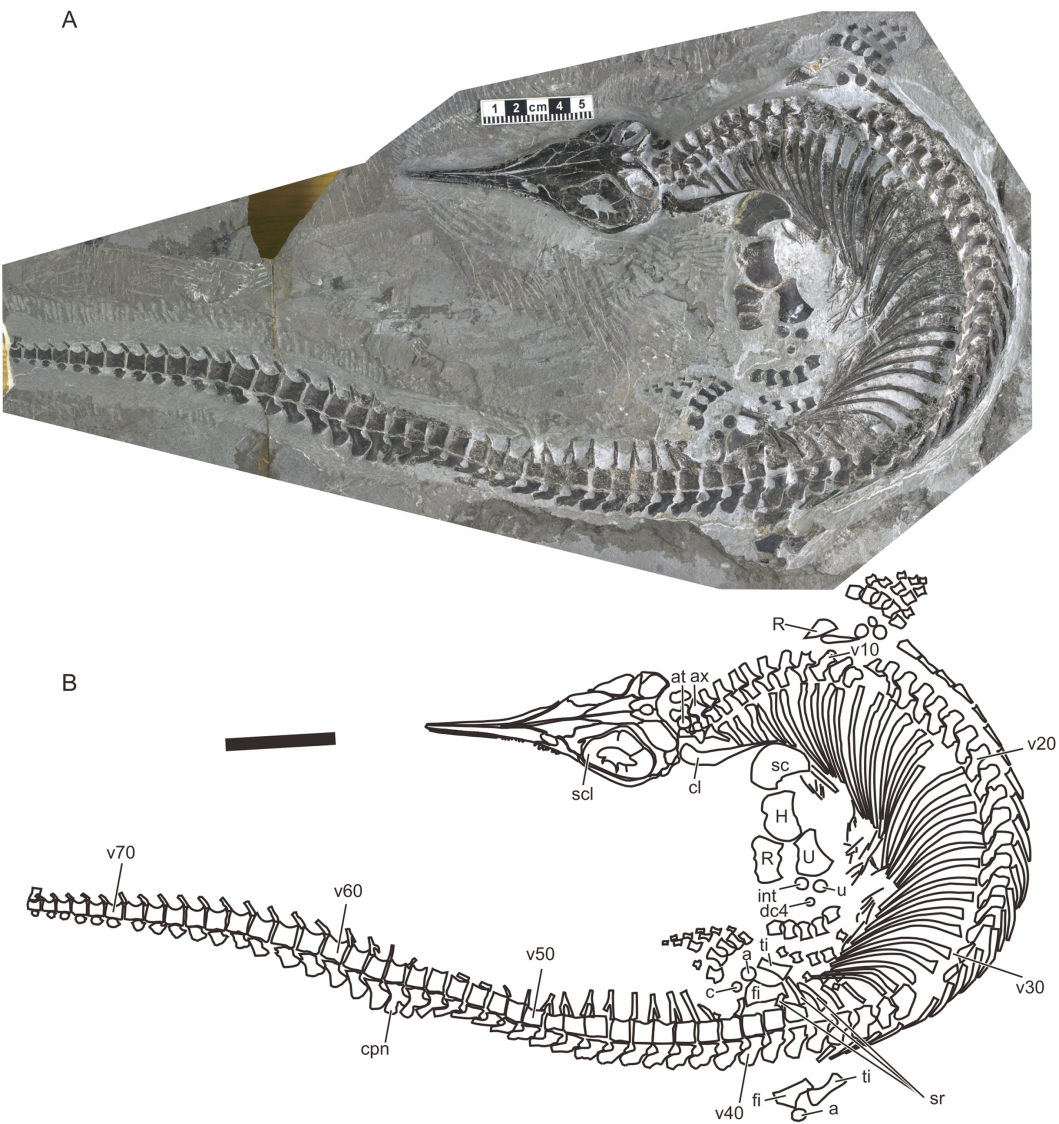
Holotype of *Chaohusaurus brevifemoralis* sp. nov (AGB7401). (A) Photograph. (B) Approximate bone map. See the section “Osteological abbreviations” for abbreviations. Scale bar is five cm in total.

A recent examination of morphological variation in these specimens revealed four morphotypes that likely represented females and males of two taxa, which were tentatively referred to as Types A and B (Motani et al., 2018). The two morphotypes were distinguished based on a suite of both qualitative and quantitative morphological characters. There are at least a dozen specimens for each morphotype—numbers that are far greater than those for the other two species of *Chaohusaurus*. These morphotypes most likely represent different species given that morphological differences do not reflect sexual dimorphism (Motani et al., 2018). Type A (Fig. 1), for which 13 specimens are known, contains the holotype of *Chaohusaurus chaoxianensis* so these specimens are considered to belong to this species. Type B (Fig. 2), with 21 specimens, had yet to be named or described, although some of the specimens were previously included in descriptive studies of *Chaohusaurus chaoxianensis* (see “Systematic Paleontology”). It is therefore important to clarify the taxonomic confusion in the existing literature. The purpose of this paper is to describe Type B as a new species of *Chaohusaurus*, and clarify its diagnostic differences with *Chaohusaurus chaoxianensis*.

## Materials and methods

### Specimens

The specimens used in this study are listed in Systematic Paleontology. Most specimens are articulated skeletons lacking some parts of the body. All specimens have undergone at least some degree of compressional deformation during preservation, adding biases to the morphological data. The degree of compression depends on the specimen. Thick bones or bony structure are most prone to the bias from such deformation. For example, when comparing specimens of similar sizes, the shaft of long bones may appear wider in one specimen than the other because of preservational deformation; the wider shaft has been flattened and widened more severely through compaction. In extreme cases, a convex surface my appear slightly concave because of compaction. The extremity of the same bones, on the other hand, seems to suffer less from similar flattening and widening, probably because the average density of the bone in these areas are higher than in the shaft. Apart from long bones, cranial structures are often distorted by compactional deformation because of their thickness. It is therefore important to compare many specimens first to grasp the original morphology of bones before compression.

### Nomenclature

The electronic version of this article in portable document format will represent a published work according to the International Commission on Zoological Nomenclature (ICZN), and hence the new names contained in the electronic version are effectively published under that Code from the electronic edition alone. This published work and the nomenclatural acts it contains have been registered in ZooBank, the online registration system for the ICZN. The ZooBank Life science identifiers (LSIDs) can be resolved and the associated information viewed through any standard web browser by appending the LSID to the prefix http://zoobank.org/. The LSID for this publication is: urn:lsid:zoobank.org:pub:3FA09089-C940-4499-ABBC-B48F0F70F38E. The online version of this work is archived and available from the following digital repositories: PeerJ, PubMed Central and CLOCKSS.

### Phylogenetic analysis

The phylogenetic position of *Chaohusaurus brevifemoralis* was analyzed based on a modified version of a published taxon-character data matrix (Motani et al., 2017). Character order has been modified to improve anatomical consistency; the correspondence between the new and old numbers are summarized in Table S1. The following characters states and descriptions were revised. A new character was added as Character 61, Dorsal orbital margin: (0) on the same plane as the rest of orbital margin; (1) with medial excursion away from the main orbital plane. The original character 68, Dentigerous region in adults, was divided into two characters, namely Character 74, Premaxillary teeth presence and Character 75, maxillary teeth presence. A new character state was added to Characters 47 and 95 respectively, to reflect the unique morphology in *Chaohusaurus* and *Cartorhynchus*—Character 47, Basipterygoid process: (0) located antero-laterally; (1) located postero-laterally and short, giving basisphenoid a square outline in dorsal view, (2) located postero-laterally and markedly expanded laterally, being wing-like, giving basisphenoid a marked pentagonal shape in dorsal view; and Character 95, Humerus anterior flange: (0) absent; (1) present but concave or notched; (2) present and complete; (3) present but reduced proximally, leaving leading edge tuberosity. State 0 of Character 108, Shape of the posterior surface of ulna, contained the word “radius” that needed to be replaced by “ulna.” Character state description of Character 177, Presacral count, was revised to: (0) less than 35; (1) between 35 and 54; (2) more than 55. These new states were incorporated into the matrix through re-coding relevant taxa. Some characters were sorted for anatomical consistency (Table S1), and the original Characters 144, Interdigital separation, and 168, Spatium interosseum between tibia and fibula size, were removed to avoid redundancy and inconsistency.

After the character revision described above, *Chaohusaurus brevifemoralis* was added to the data matrix and coded. The following characters in other Early Triassic taxa were recoded to reflect updated information through direct observations and the literature. The new coding are: *Cartorhynchus lenticarpus*, 36(1), 37(1), *Chaohusaurus geishanensis*, 22(1), 36(1), 59(0/1), 63(0); *Chaohusaurus chaoxianensis*, 22(1), 26(1), 28(1), 36(1), 37(1), 42(?), 46(?), 59(0/1), 63(0), 71(?), 93(1), 119(1), 172(?), 173(0), 174(0), 196(0), 197(0); *Chaohusaurus zhangjiawanensis*, 26(1), 28(?), 37(1), 116(0), 135(0); *Hupehsuchus nanchangensis*, 34(0). Also, dental characters were recoded for nasorostrans, and Characters 168, Spatium interosseum between tibia and fibula presence, and 169, Hind fin leading edge element in adults, were recoded to comply with a previous study (Ji et al., 2016). The revised character matrix is provided in Data S1.

The resulting character-taxon matrix was analyzed by TNT 1.5 (Goloboff, Farris & Nixon, 2000) and PRAP2 (Müller, 2004) with PAUP* 4b10 (Swofford, 2003). Searches in TNT used a combination of options defined by “xmult = hit 100 replications 100 drift 10 hold 10.” Searches in PRAP2 were based on default setting of 200 replicates of rachet searches. The Bremer support values were calculated in TNT, using a command “bsupport !!+0 1.” Bootstrap values were also calculated in TNT, based on 10,000 replicates.

### Sexing

We are not providing new results on sexing of the specimens over what were recently published (Motani et al., 2018). However, it may be useful to summarize the rationale behind the published sexing results that are adopted in the present paper. Motani et al. (2018) found that two taxa, herein identified as *Chaohusaurus chaoxianensis* and *Chaohusaurus* *brevifemoralis*, each contained two morphotypes with long and short limbs, respectively. Such intraspecific dimorphism in relative limb length to the body is commonly known in multiple marine tetrapods, where males have longer limbs than females but never the other way around. This phenomenon is rationalized by the notion of the “organ of prehension” laid out by Darwin. That is, males usually have organs to hold females during courtship, which he called “organ of prehension,” and elongated limbs often evolve to serve that purpose. Based on this rationale, Motani et al. (2018) identified the morphs with elongated limbs as males. See Motani et al. (2018) for the details.

### Statistical analysis

As stated earlier, the purpose of the present paper is purely to describe a new species that was previously established as a distinctive morphotype based on both qualitative and quantitative characters, aided by statistical analyses (Motani et al., 2018). It would be repetitive to report the analyses again, so the readers are advised to refer to that paper for the statistical backgrounds.

## Systematic paleontology

Ichthyosauromorpha Motani et al., 2015a
Ichthyosauriformes Motani et al., 2015a
*Chaohusaurus* Young and Dong 1972
*Chaohusaurus chaoxianensis* (Chen, 1985)

*Anhuisaurus chaoxianensis*, Chen, 1985

*Anhuisaurus faciles*, Chen, 1985

*Chensaurus chaoxianensis* Mazin et al. 1991

*Chensaurus faciles* Mazin et al. 1991

*Chensaurus chaoxianensis* Motani and You 1998a, in part

*Chaohusaurus geishanensis* Motani and You 1998b, in part

*Chaohusaurus chaoxianensis* Motani et al. 2015c, in part

**Holotype.** AGM AGB2905 (previously referred to by a field number P45-H85-25).

**Paratype.** P45-H85-24 (whereabouts unknown).

**Referred specimens.** AGM AGB2906 (P45-H85-20, holotype of *A. faciles* Chen, 1985), 5855, 6252, 6259, 6261, 6262, 6608, 6609, 7404, 7409, 7413; NGM P45-H85-21, P45-H85-23 (paratype of *A. faciles* Chen, 1985); IVPP V11362.

**Figure 3:**
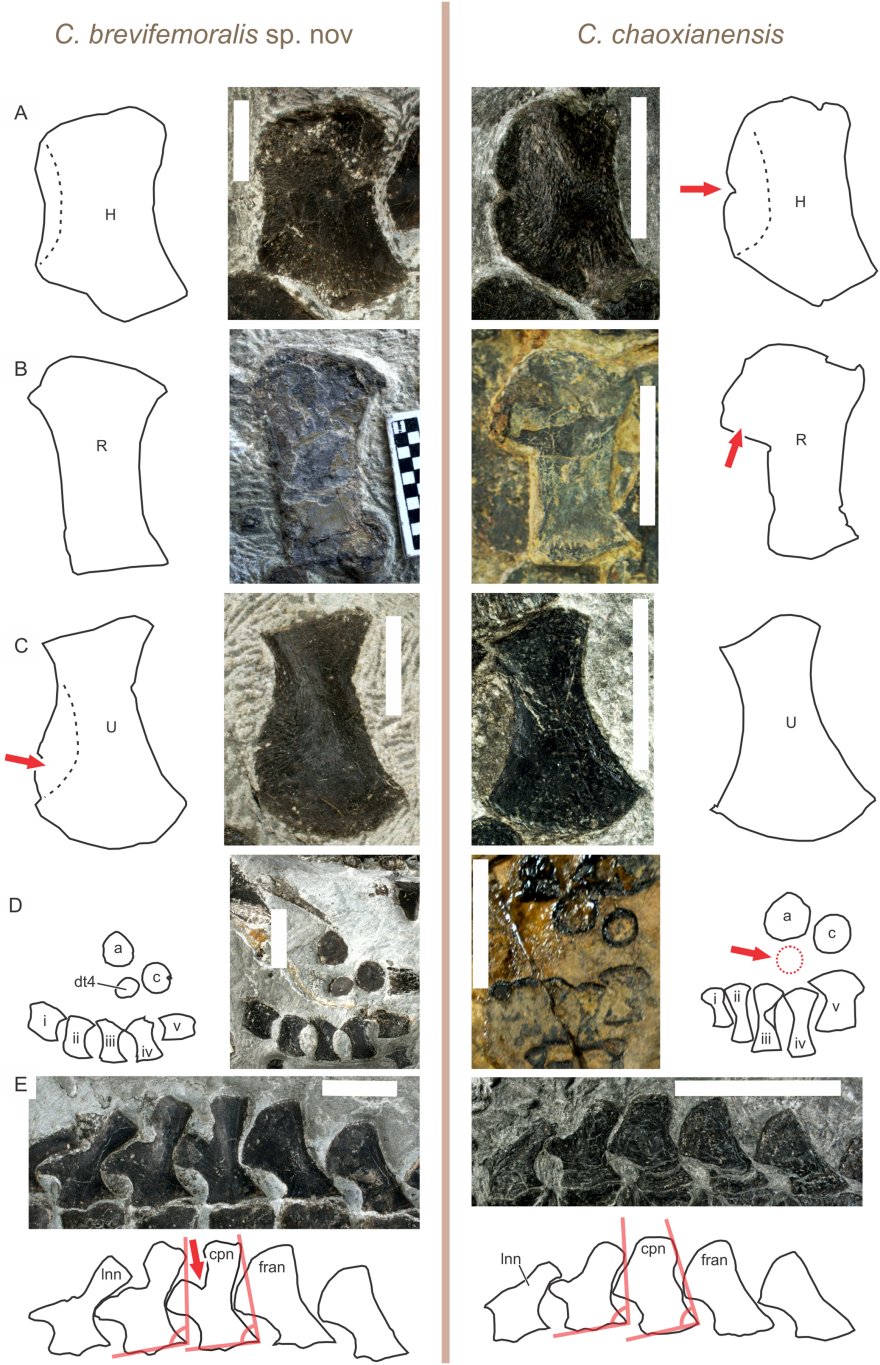
Comparison of diagnostic features between *Chaohusaurus brevifemoralis* sp. nov and *C. chaoxianensis*. (A) Anterior flange of the humerus, which is poorly developed in *C. brevifemoralis* (AGB 6258) but well-developed in *C. chaoxianensis* (AGB 6256) to leave a notch in the middle. (B) Anterior flange of the radius, which is poorly developed in *C. brevifemoralis* (AGB 7403, paratype) but well-developed in *C. chaoxianensis* (AGB 2905, holotype). (C) Anterior expansion of the distal ulnar shaft, which is present in *C. brevifemoralis* (AGB 6260) and makes the distal fan of the ulna appear asymmetrical relative to the ulnar axis (note: usually smaller than in the specimen figured here) but absent in *C. chaoxianensis* (AGB 6262), leaving the distal fan appear symmetrical relative to the ulnar axis. (D) Distal tarsal, at least one of which is always present in *C. brevifemoralis* (AGB 7401, holotype) but is absent in most specimens of *C. chaoxianensis* (AGB 2905, holotype) except the largest individuals. (E) Caudal peak neural spine (middle one of the five figured), which is bifurcated in *C. brevifemoralis* (AGB 7401, holotype) but remains single in *C. chaoxianensis* (AGB 5855). Red arrows point to apomorphic character states. Orange lines in (E) describe the inclination angles used in identification of the first anticlined neural spine explained in text. Scale bars are one cm.

**Revised diagnosis.** Narrow notch between two anterior sub-flanges of humerus; antero-proximal flange of radius well differentiated from shaft; ulnar distal fan nearly symmetrical relative to bone axis; only two tarsal ossifications in most individuals except largest; caudal peak neural spine not bifurcated (Fig. 3).

**Remarks.** The caudal peak neural spine of the holotype may appear bifurcated due to a damage. However, the impression left on the matrix suggests that it was not bifurcated in life. The morphotype representing this species was referred to as Type A by Motani et al. (2018).

*Chaohusaurus brevifemoralis* sp. nov.

*Chensaurus chaoxianensis* Motani and You 1998a, in part

*Chaohusaurus geishanensis* Motani and You 1998b, in part

*Chaohusaurus chaoxianensis*, Motani et al. 2015c, in part

*Chaohusaurus chaoxianensis*, Zhou et al. 2017, in part

**Etymology.** The specific name refers to the shortness of the femur relative to the body in comparison to other species.

**Figure 4:**
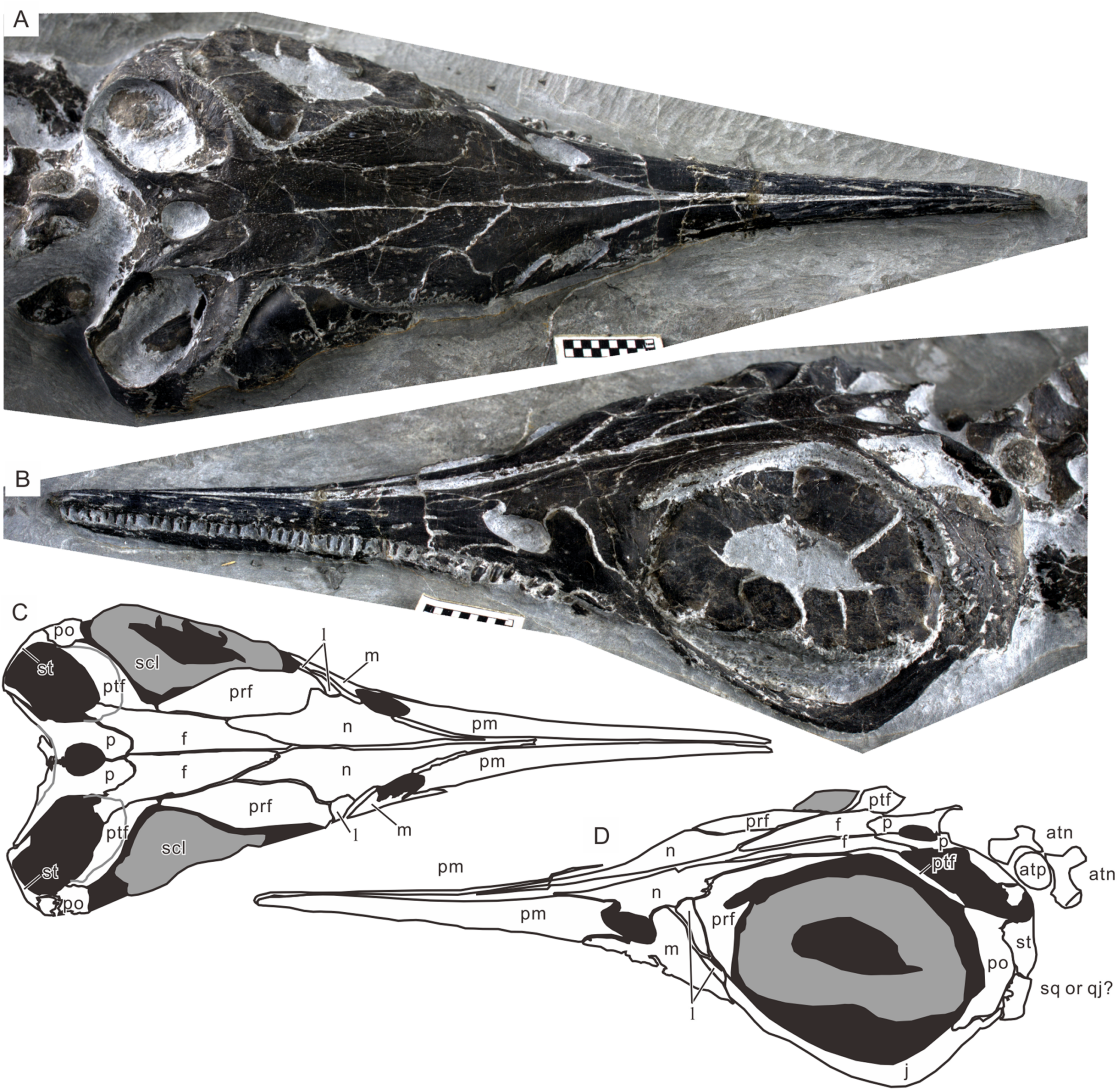
Skull of the holotype of *Chaohusaurus brevifemoralis* sp. nov (AGB7401). (A) Dorsal view. (B) Lateral and slightly dorsal view. (C) Approximate bone map for (A). (D) Same for (D). See the section “Osteological abbreviations” for abbreviations. Scale bar is one cm in total.

**Holotype.** AGM AGB7401. A complete skeleton of a male individual, lacking only the tip of the tail. From bed 621. See Figs. 2 and 4.

**Figure 5:**
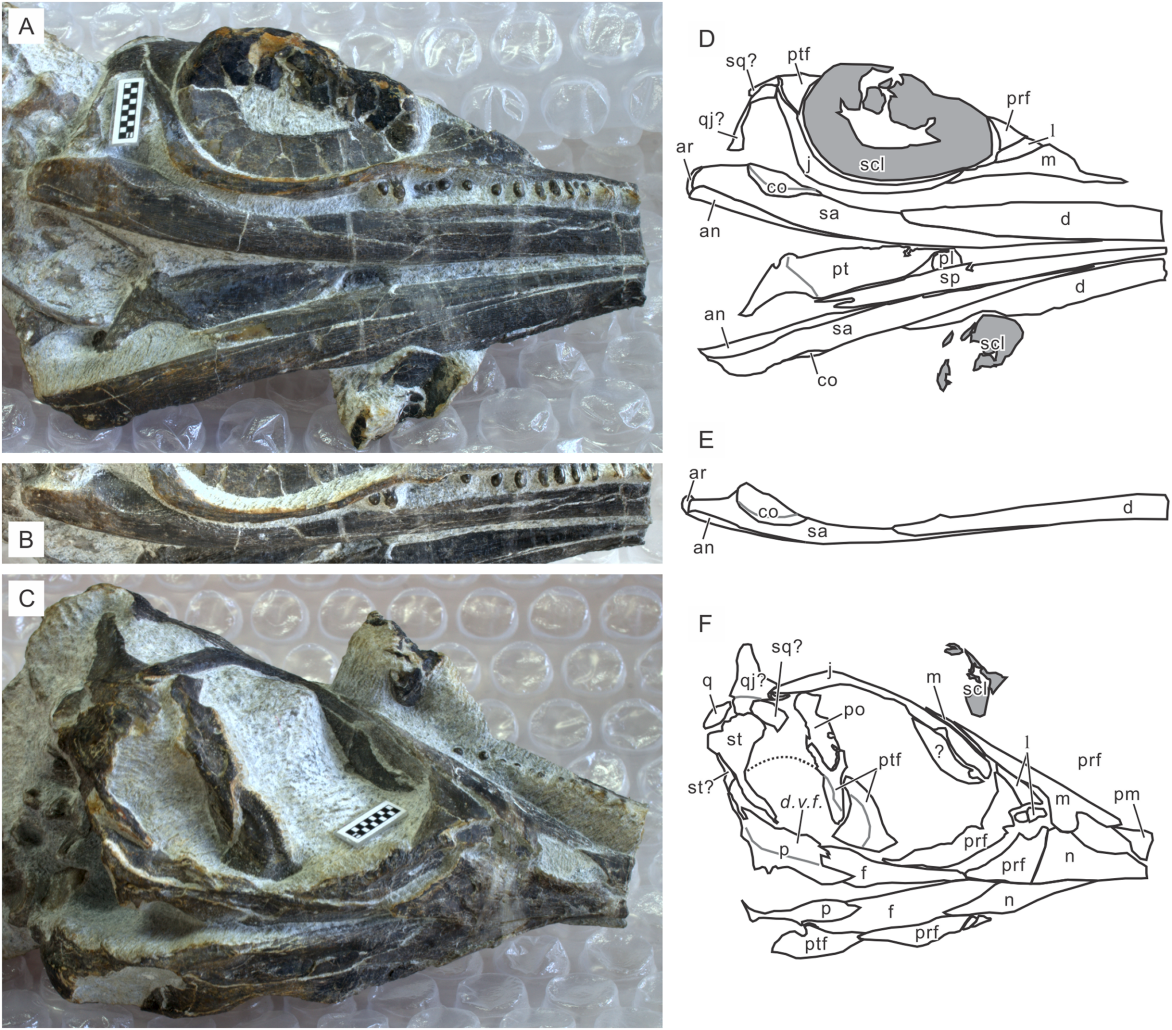
Skull of *Chaohusaurus brevifemoralis* sp. nov in one of the paratypes (GMPKU P-3086). (A) Ventral view of the mandible and lateral view of the right side of the skull. (B) Right mandibular ramus from dorso-lateral direction. (C) Approximately lateral view of the left side of the skull. (D) Approximate bone map for (A). (E) Same for (B). (F) Same for (C). See the section “Osteological abbreviations” for abbreviations. Scale bar is one cm in total.

**Figure 6:**
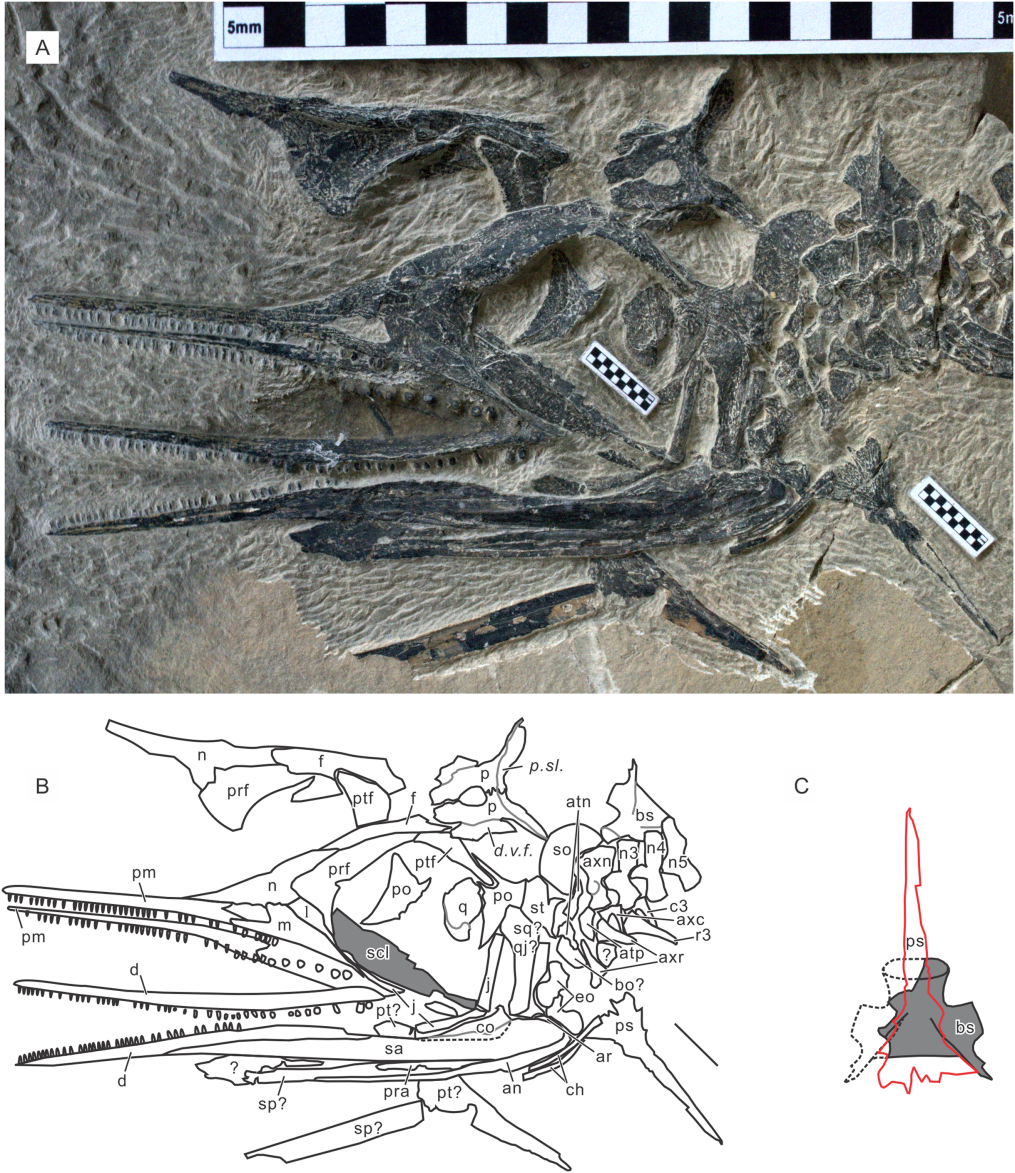
Skull of *Chaohusaurus brevifemoralis* sp. nov in one of the paratypes (AGB7403). (A) Planar view of the specimen. (B) Approximate bone map for (A). (C) Reconstruction of the parasphenoid-basisphenoid complex. See the section “Osteological abbreviations” for abbreviations. Short scale bars are one cm each.

**Paratypes.** GMPKU P-3086. A nearly complete skeleton of a female individual lacking the tip of the snout and most of the forelimb and pedal phalanges. From bed 633. The specimen was recently figured (Zhou et al., 2017). See also Fig. 5. AGB7403. A partially disarticulated skull and the upper body down to the forelimb (Fig. 6). From bed 628.

**Referred specimens.** AGM AGB5846a, 5846b, 5846c, 6253, 6254, 6255, 6258, 6260, 6605, 7402, 7407, 7408, 7410, MT10022; GMPKU P-1101, P-3093; IVPP V11361.

**Ambiguous specimens.** AGB6607 and 7400 are not included in the formal list of the specimens but may belong to *Chaohusaurus brevifemoralis*. As reported elsewhere (Motani et al., 2018), both share the same quantitative scaling trends and qualitative features with *Chaohusaurus brevifemoralis*. Yet, they appear osteologically immature compared to specimens of the same sizes, that is, their bones are slender, and extremities of long bones are not fully formed. At least one of them (AGB6607) is large enough to be an adult of *Chaohusaurus brevifemoralis*. Based on this inferred immaturity, we are not including them in the list of referred specimens at this point.

**Locality.** Majiashan, Chaohu, Anhui Province, China.

**Horizons.** Ammonoid *Subcolumbites* zone, Spathian, Lower Triassic. Known specimens are from beds 621 to 638 (about 248.53–248.34 Ma) that were previously dated using astrochronology (Fu et al., 2016).

**Figure 7:**
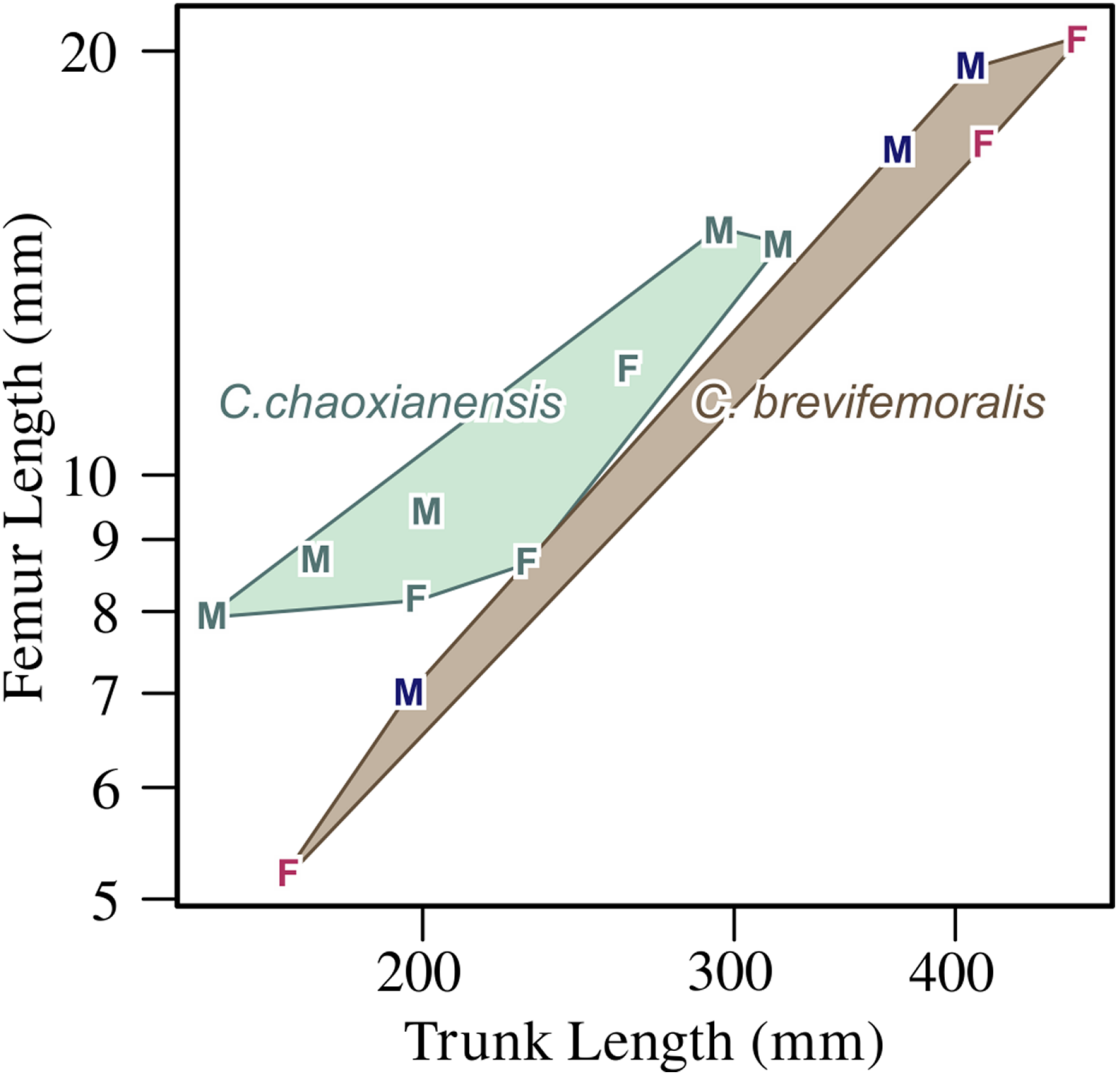
Femoral length plotted against trunk length. F, female; M, male. Within each species, males tend to have longer femora for the trunk length than females, while *C. chaoxianensis* tends to have longer femora for the trunk length than *C. brevifemoralis*, especially when compared within the same sex. Polygons represent convex hulls for each species. Based on Motani et al. (2018).

**Diagnosis.** Humeral anterior flange poorly developed, with weakly concave preaxial margin near midshaft; radial antero-proximal flange poorly developed; ulnar distal fan asymmetrical relative to bone axis, due to anterior expansion of distal preaxial margin; femur short for trunk length in comparison to *Chaohusaurus chaoxianensis*; tibia proximally narrow for trunk length in comparison to *Chaohusaurus chaoxianensis*; bifurcated neural spine near caudal peak; three tarsal ossifications in most individuals except newborns (Figs. 3 and 7).

**Remarks.** The morphotype representing this species was established as Type B by Motani et al. (2018). Quantitative and qualitative comparisons with *Chaohusaurus chaoxianensis* (Type A) are found in that paper.

## Results

### Morphological descriptions

The body of *Chaohusaurus brevifemoralis* sp. nov is usually preserved in a curled posture, as seen in the holotype (Fig. 2). This tendency for curled posture is shared with *Chaohusaurus chaoxianensis* (Fig. 1), and probably with other species of the genus. To the best of our knowledge, there are only two specimens of *Chaohusaurus* that are preserved in approximately straight posture, one of which was previously figured (Motani, You & McGowan, 1996).

The body proportion in adults is similar to those of non-hueneosaur ichthyopterygians—the tail occupies about half of the total length, and the skull is usually about a quarter of the precaudal length (i.e., about one eighth of the total length). The relative size of the skull to the total length is much larger in long-snouted hueneosaur ichthyopterygians, where the values scatter around 20–30%.

The sizes of the major structures in the holotype and four more specimens are summarized in Table 1 and compared to the same metrics for four specimens of *Chaohusaurus chaoxianensis*. To avoid redundancy, absolute sizes are not reported in text.

**Table 1:** Measurements of major structures in *Chaohusaurus brevifemoralis* in females and males of different sizes.

Species	*C. brevifemoralis*					*C. chaoxianensis*				
Institution	GMPKU	AGM	AGM	GMPKU	AGM	AGM	AGM	AGM	AGM	AGM
Specimen number	P-1101	AGB7401	AGB6260	P-3086	AGB7408	AGB2906	AGB2905	AGB6259	AGB6262	AGB6456
Sex	F	M	M	F	F	F	F	M	F	M
Length along the vertebral column		(709+)	(816+)						542	505
SVL	238.9	442	529		578				305	288
Trunk length	168	334	408.1	415.3	469	136.6	261.7	294.13	229.38	201
Presacral vertebral count		36			37			36	35	36
Sacral vertebral count		3								
Preserved caudal vertebral count		(36+)								
Skull length	70.86	114.61	120.9		111.75	58.97			75.62	87
Orbit length	20.71	35.07		25.82	32.57		25.73			26.1
Scleral ring aperture length		16.2								
UTF maximum diameter		15.3		(18.49−)						
External naris length		8.63			7.53				5.18	6.01
Humerus length	7.02	23.22		18.7	25.59	6.97	19.56	20.38	14.03	16.5
Humerus distal width	6.04	15.72		13.4	20.53	5.27		15.44	11.2	10.04
Radius length	7.74	21.69	24.02	16.32	20.96	6.64	15.53	18.35	13.94	13.5
Radius proximal width	4.76	14.71	13.34	11.42		4.54	10.42	13.34	9.18	9.38
Ulna length	7.94	20.52	24.79		22.31	6.87	15.38	17.46	14.03	12.46
Ulna distal width	3.68	15.98	11.88		16.47	4.77	8.36	13.84	9.51	7.35
Intermedium maximum diameter	1.29	6.38	6.17		7.66		0.89	6.05	4.54	3.39
Femur length	5.22		19.38	17.2	20.39		11.9	14.93	8.68	9.43
Femur distal width	4.75		13.88	10.32	15.81		8.17	10.47	6.73	9.21
Tibia length	6.01	17.99		15.93			10.14	14.38	9.61	9.87
Tibia proximal width	3.42	6.98		7.06			5.5	7.28	5.38	4.36
Fibula length	7.38	19.13	20.12	18.01			11.42	15.84	10.25	10.02
Fibula distal width	4.4	12.01	14.22	12.06			7.61	10.78	7.48	7.01
Astragalus maximum diameter	1.49	6.66		6.77			3.92	4.96	4.1	3.62
Specimen note	Smallest	Holotype	Largest M	Paratype	Largest F	Smallest	Holotype	Largest M	Typical F	Typical M

#### Cranium

Cranial suture patterns are given in Figs. 4–6, so the contact patterns among bones are minimally reported in the description below to save space. Exceptions are bones surrounding major fenestrae, and ambiguous or polymorphic cases. Unless otherwise stated, descriptions of cranial and mandibular elements below are based on the holotype (Fig. 4) and the paratypes (Figs. 5 and 6), one of which revealing some hidden parts of cranial elements through partial disarticulation.

**Premaxilla.** The premaxilla is a slender bone that occupies most of the snout in lateral view, where its length is slightly less than half of the skull length. Its extent along the midline is less because the nasals extend anteriorly in-between the two premaxillae. The premaxilla is approximately triangular but has short sub- and supranarial processes emerging from the ventral and dorsal corners of the posterior margin, which forms the anterior margin of the external naris. One of these processes may be missing in some specimens without a clear pattern. The premaxilla bears teeth, as described below.

**Maxilla.** The maxilla is about half as long as the premaxilla along the jaw margin. It has an ascending postnarial process that excludes the lacrimal from the margin of the external naris. This process is robust, with its base width rivaling the antero-posterior length of the external naris. The suture with the lacrimal appears smooth. The maxilla forms the posterior and postero-ventral margins of the external naris, forming a continuous curve that contributes to about a third of the oval perimeter of the opening. The anterior extent of the maxilla in lateral view is slightly anterior to the external naris, while the posterior end of the bone in lateral view overlaps the anteriormost part of the orbit narrowly. The maxilla bears teeth, as described below.

**Nasal.** The nasal is a major bone that forms a part of the skull roof from the anterior orbital to prenarial region, behind the major part of the snout. It is widest near the lacrimal, and tapers to a point both posteriorly and anteriorly. The posterior ends of the right and left nasals are widely separated by the frontals while the anterior ends meet along the sagittal line. In the holotype, the right nasal appears to extend far more anteriorly than the left nasal, but this is likely an artifact of preservation (Figs. 4A and 4C).

**Frontal.** The frontal is a major bone that forms the skull roof in the orbital region. Its shape is approximately an elongated triangle in dorsal view. Its postero-lateral process narrowly participates in the antero-lateral margin of the upper temporal fenestra, forming the most medial segment of the anterior terrace of the fenestra (Fig. 4). The bone is excavated in the terrace region, being separated from the roof part by a conspicuous ridge. The ridge and terrace are confluent with the corresponding structures of the postfrontal.

**Parietal.** The parietal has the smallest exposure of the bone of the skull roof, comprising the main corpus forming the posteriormost part of the skull roof and a supratemporal process that extends postero-laterally from the corpus. The corpus appears small compared to those of most ichthyopterygians in its relative size to the skull, in width and length. There is a ridge at the posterior end of the skull roof, followed by a slope that is better seen in occipital view than dorsally. They likely represent the parietal ridge and slope that are present in mixosaurs and parvipelvians (Motani, 1999). The inter-parietal suture is not straight: it is bent once in front of the pineal foramen and deeply interdigitated posterior to the foramen (Figs. 4 and 6). It is evident that there is a descending ventral flange along the margin of the upper temporal fenestra, as expected in neodiapsids. The flange is separated from the roof by a conspicuous ridge.

**Postfrontal.** The postfrontal forms a major part of the bar between the orbit and upper temporal fenestra. It is posterior two-thirds are excavated to form most of the anterior terrace of the upper temporal fenestra (Fig. 4). This proportion of excavated area is unusually high compared to ichthyopterygians. The excavated terrace is separated from the skull roof by a sharp and conspicuous ridge that continues into the frontal.

**Prefrontal.** The prefrontal is the largest of the bones surrounding the orbit (Figs. 4–6). It has a structure often referred to as an “eyebrow,” a small shelf above the antero-dorsal corner of the orbit. This feature is shared with basal ichthyopterygians, such as *U. hataii* and *Grippia longirostris* (Mazin, 1981; Motani, Minoura & Ando, 1998; Motani, 2000; Cuthbertson, Russell & Anderson, 2013b). The bone is swollen laterally around the base of this structure, making it appear unusually massive for a cranial dermal bone.

**Lacrimal.** The lacrimal appears as a narrow strip of bone between the maxilla and prefrontal in lateral view (Figs. 4 and 5). It is wider away from the orbit. See Orbit below for its participation in the structure.

**Jugal.** The jugal is a narrow and J-shaped bone in lateral view (Figs. 4–6) but there is indeed much three-dimensionality to the morphology of the bone. The bone is most robust near the postero-ventral corner, where it has a thick and rounded cross-section. From there extends a narrow and horizontal plate of bone anteriorly, forming the maxillary process along the antero-ventral margin of the orbit. This plate widens anteriorly along the orbital rim. This ventral margin of the orbit formed by the jugal is continuous with the anterior wall of the orbit formed by the prefrontal, together giving depths to the orbital rim. Postero-dorsal to the postero-ventral corner extends the postorbital process. Unlike the maxillary process that is flat horizontally, the postorbital process is flat vertically. The postorbital process articulates mainly with the postorbital but it also contacts the quadratojugal and may be partially overlapped by the squamosal.

**Postorbital.** The postorbital is best seen in AGB7403, where the bone is disarticulated from the rest of the skull (Fig. 6). Its general outline may be compared to the bottom half of the waxing crescent. There are two short processes extending antero-dorsally (postfrontal process) and postero-dorsally (supratemporal process), respectively.

**Supratemporal.** The supratemporal is a large bone that forms the postero-lateral corner of the upper temporal fenestra. It resembles the squamosal of neodiapsids. it is essentially a triradiate bone, with the postorbital process extending anteriorly, parietal process medially, and descending process ventrally. The descending process is excavated to allow acceptance of the dorsal end of the quadrate, as in *Ichthyosaurus* (McGowan, 1973).

**Squamosal.** The identity of squamosal and quadratojugal requires future scrutiny because some specimens show just one of them each. The following description is based on the tentative identification given in Figs. 4–6 with question marks. The squamosal in *Chaohusaurus* spp. resembles that described for *Mixosaurs atavus* in that it is slightly swollen laterally and occupies the middle part along the height of the cheek, unlike in most diapsids where it is located dorsally. The bone covers parts of the supratemporal and quadrate superficially, and sometimes also parts of the postorbital and jugal. The bone appearsmissing in some specimens, possibly reflecting its superficial position that makes it easy for the bone to flake off. However, analyses of 3D morphology are necessary to clarify if the bone is truly missing in these specimens. This bone was identified in several different locations and with different shapes depending on the specimen in a previous publication (Zhou et al., 2017). One of them, figured in their Fig. 4B, matches the current description (see “Discussion”).

**Quadratojugal.** Again, the identity of squamosal and quadratojugal requires future scrutiny because one of the bones may not be clearly present in some specimens (see “Squamosal”). The quadratojugal is a vertically elongated plate of bone that is slightly broader dorsally than ventrally. It is constricted above the ventral end, where the bone is expanded and thickened to form a cup that sits on the dorso-lateral part of the quadrate condyle, as in *Ichthyosaurus* (McGowan, 1973).

**Quadrate.** The outline of the quadrate in postero-medial view may be compared to that of a human auricle, with the quadrate condyle occupying the position of the lobule of the auricle. This shape is most evident in the right element in AGB7403 that has been disarticulated and exposed in postero-medial view (Fig. 6). The condyle has two ridges separated by a shallow groove, as in most reptiles (Romer, 1956). When in articulation, the bone is largely concealed by the quadratojugal that covers most of its lateral aspect, and the supratemporal that encloses its dorsal end.

**Supraoccipital.** The supraoccipital is missing in the holotype but exposes its postero-dorsal view in AGB7403, being disarticulated from but associated with the parietals (Fig. 6). It is a large bone for the occiput, being slightly larger than the roof part of a single parietal. Its antero-dorsal margin is convex, and postero-ventral margin concave. The lateral margins are approximately straight. The shape of the convex margin approximately fits that of the concave posterior margin of the parietal pair, to which it is supposed to articulate. No articular facets are seen on the exposed side, as expected from the condition in other ichthyosauriforms.

**Exoccipital.** There is a pair of exoccipitals preserved behind the skull of AGB7403, in disarticulation (Fig. 6). The exoccipital resemble that described for *Ichthyosaurus* in having a shape somewhat reminiscent of a boot in lateral view; it is wider ventrally than the dorsally, the posterior margin is approximately vertical, and the anterior margin is strongly concave (McGowan, 1973). The toe of the boot is conspicuously curled-up in *Ichthyosaurus*, but the curling is not as pronounced in the present form.

**Basioccipital.** The basioccipital of *Chaohusaurus* in general is largely unknown. In AGB7403, there is a bone between the skull and the atlantal pleurocentrum that may represent the basioccipital, being exposed in what appears to be the dorsal view given the presence of a pair of articular facets, probably for the exoccipitals (Fig. 6). The bone is longer than the atlantal pleurocentrum antero-posteriorly, and its length approximately matches the distance between the posterior margins of the parasphenoid and basisphenoid that will be mentioned below.

**Basisphenoid.** The basisphenoid is known from only one specimen, AGB7403, which exposes more than half the bone, dislocated to be behind the supraoccipital (Fig. 6). There is no sign of a major foramen in the exposed side, so it is most likely the ventral side. Figure 6C represents the estimation of the bone outline through mirroring of the exposed half. The reconstructed shape is reminiscent of what was drawn for *Limnoscelis* (Romer, 1956). Unlike in *Ichthyosaurus*, the basisphenoid of *Chaohusaurus* is not fused to the parasphenoid. There is a pair of processes pointing postero-laterally from the posterior corners of the bone, and this process continues ventrally to the main body of the bone where they become side walls of a triangular area that is excavated from the ventral side. This area seems to serve as the socket for the main body of the parasphenoid. The triangular area matches the shape of the main body of the parasphenoid (Fig. 6C). Conspicuous striations are seen lateral to the triangular area, resembling those that are reported for the parasphenoid below. The basipterygoid process is short, broad, and directly laterally, not antero-laterally as in most reptiles. The anterior surface of the basisphenoid is slightly concave.

**Parasphenoid.** The parasphenoid is best seen in AGB7403 (Fig. 6). It is a large bone that covers the base of the braincase region, comprising a long cultriform process and a fan-shaped main body. The anterior half of the fan fits into a triangular socket at the ventral part of the basisphenoid, while the posterior half extends more posteriorly than the main body of the basisphenoid, almost reaching the tip of the postero-lateral process of the basisphenoid (Fig. 6C). The main body is covered by strong striations that radiate from the base of the cultriform process. When articulating the parasphenoid to the basisphenoid, these striations would become a part of a series with the striations reported above for the basisphenoid. There is a pair of swelling at the base of the cultriform process, which are probably basal tubera (Romer, 1956).

**Pterygoid.** The pterygoid is best seen in GMPKU P-3086 (Fig. 5A). It is similar to the pterygoid of basal ichthyopterygians, such as *Grippia longirostris*. There is a transverse flange marked by a weak ridge that runs from postero-medial to antero-lateral. Pterygoidal teeth are not present. The part posterior to this ridge is elevated and then becomes slightly twisted toward the posterior end, forming the quadrate ramus. The anterior part tapers to a point along the sagittal plane. As with the pterygoid, there is no sign of the margin for the suborbital fenestra.

**Palatine.** GMPKU P-3086 has a part of the palatine exposed, showing that it is located in its usual position in ichthyopterygians, that is, antero-lateral to the anterior ramus of the pterygoid (Fig. 5A). There is no evidence of the suborbital fenestra in the exposed part of this bone. Otherwise, the exposure is too limited to allow morphological descriptions.

**Orbit.** The orbit is large and occupies a substantial part of the postnarial skull, with a maximum diameter that is about twice as large as that of the upper temporal fenestra. The orbital rim does not form a circle, ellipse, or ovoid that would fit onto a plane as in ichthyosaurians. Instead, the rim exhibits a conspicuous medial excursion in the dorsal part, off the lateral plane formed by the remainder of the rim (Figs. 4A and 4C). The excursion corresponds to the constriction of the skull roof near the narrow contact between the prefrontal and postfrontal. Similar constriction of the skull roof and an associated excursion of the orbital rim have been recognized in *Grippia longirostris*, *U. hataii* and *Cartorhynchus lenticarpus* (Motani, Minoura & Ando, 1998; Motani, 2000; Motani et al., 2015a) and seems to be a basal ichthyosauriform feature. The excursion makes the eyeball vulnerable to stresses from the dorsal aspect. This vulnerability is partly remedied by the extensive growth of the scleral ring, as mentioned below.

The orbital rim is formed by the prefrontal anteriorly and antero-dorsally, postfrontal postero-dorsally, postorbital posteriorly, jugal ventrally, and slightly by the lacrimal antero-ventrally. The participation by the lacrimal may not be evident in Fig. 4D but it is largely because of the posterior side of the prefrontal being visible in lateral view, making the orbital rim smaller than it is. It is evident in AGB7403 (Fig. 6). The frontal is excluded from the orbital rim by a narrow contact between the pre- and postfrontals.

**External Naris.** The normal axis for the planed formed by the margin of the external naris points dorso-laterally and slightly anteriorly, being directed more dorsally than laterally in the holotype. The opening is elongated, and surrounded by the maxilla ventrally and posteriorly, nasal dorsally, and premaxilla anteriorly. The lacrimal is completely excluded from the margin, as in basal ichthyopterygians.

**Upper Temporal Fenestra.** The upper temporal fenestra extends from antero-medial to postero-lateral direction, with its parasagittal extent being less than the lateral extent (Fig. 4). The skull of *Chaohusaurus* does not appear narrow despite the smallness of the parietals thanks to the wide upper temporal fenestra. It is bordered by the parietal medially and posteromedially, supratemporal posterolaterally, postorbital antero-laterally, postfrontal anteriorly and slightly by frontal anteromedially. There is no evidence for the squamosal to participate in the margin of the fenestra.

**Lower Temporal Fenestra.** The lower temporal fenestra is a prominent structure in the cheek that is vertically elongated and approximately wedge-shaped. It is completely open ventrally. This morphology is shared with basal ichthyosauromorphs, including *Cartorhynchus lenticarpus* and Hupehsuchia (Chen et al., 2014c; Motani et al., 2015a; Wu et al., 2016), but not with ichthyopterygians.

**Pineal Foramen.** The pineal foramen is completely enclosed between the right and left parietals. Its center is located posterior to the midpoint along the inter-parietal suture. Its shape is ovoid, being wider posteriorly than anteriorly. It is located behind the line connecting the posterior margins of the orbits in the holotype, but the location may differ depending on the direction of compaction experienced by relevant specimens.

**Scleral ring.** In parvipelvian ichthyosaurs, the scleral ring has a central part that is shaped like the wall of a low conical frustum, with the aperture corresponding to the top of the frustum, and a peripheral part that continues from the base of the frustum to form a round wall around the axis of the central frustum (which is expected to be nearly parallel to the optical axis of the eyeball) (Motani, 2005). The central frustum and peripheral wall form an angle of about 110° to 130° (Motani, 2005; Moon & Kirton, 2016). The center of the frustum part may protrude beyond the plane formed by the orbital margin in life. The central part, however, is preserved flattened in most specimens because of preservational compaction, and appear as a flat ring rather than the wall of a conical frustrum. The scleral ring is very thin and deformable even in extant specimens of reptiles, and can easily change its shape plastically through preservation.

This basic arrangement is already present in *Chaohusaurus brevifemoralis*, although the peripheral wall appears shorter in the present form than in Jurassic forms. The right scleral ring of the holotype suggests that the central frustum formed an angle of about 120° with the peripheral wall, giving depth to the scleral ring. The left ring has a flattened central part, making it appear as if the central part was completely parallel to the plane formed by the orbital margin, but this is most likely an artifact of preservation as in many parvipelvian specimens. The scleral ring is the largest bony structure in the cranial region, apart from the entire skull itself, filling the large orbit (Fig. 4). The aperture of the ring is also large. A part of the dorsal aspect of the ring is exposed through the constriction of the skull where the medial excursion of the orbital rim occurs. It is seen there that the peripheral wall of the scleral ring fills the embayment of the skull roof (Figs. 4A and 4C), solidifying the eyeball that would otherwise lack bony support in the dorsal direction. The ring is inclined, with the normal axis pointing mostly laterally but also tilted anteriorly and dorsally to some extent. The left scleral ring of the holotype seems to comprise about 16 plates, although it is difficult to arrive at the exact number because the preservation is not perfect. There seem to be 18 plates in the right scleral ring of GMPKU P-3086 but the number, again, is not conclusive.

#### Mandible

The mandible most likely comprises seven elements, including the coronoid that is lacking in derived ichthyosaurs (McGowan, 1973). The mandibular rami are slightly curved and inclined in natural posture, with the anterior part more vertical than the posterior part that is half horizontal. The lateral side of the mandible appears flat in specimens with disarticulated jaws but this is because of flattening by compaction.

**Dentary.** As with ichthyopterygians, the dentary is an elongated bone that is longer than half the length of the mandible. It is the only dentigerous bone in the lower jaw. The presence of a dental groove is not clearly established because the medial side of the dentary is rarely exposed. However, it is seen in AGB7403 that the teeth are medial to a bony wall, as seen in subthecodont ichthyopterygians. See below for its dentition.

**Surangular.** The surangular is the most prominent bone of the mandible in lateral view, occupying most of the posterior half of the structure (Figs. 4 and 6). It extends anteriorly beyond the anterior margin of the external naris. Posteriorly, it supports the lateral aspect of the articular, which forms the jaw articulation with the quadrate. There seems to be no direct contact with the quadrate.

**Angular.** The angular is an elongated bone that covers the ventral aspect of the mandible, from the posterior end to about the midpoint. It is visible both laterally and medially. However, its lateral exposure is limited, unlike in *Cartorhynchus lenticarpus* and many basal neodiapsids where it occupies the ventral half or more of the posterior part of the lateral surface (Motani et al., 2015a).

**Articular.** The articular is barely visible in lateral view, although its dorsal margin may be seen as a narrow band surrounding the poster-dorsal corner of the surangular in some specimens (Fig. 6). The articulation with the quadrate condyle seems to occur with the postero-medial surface of the articular, unlike in *Ichthyosaurus* where the articulation is through the antero-medial surface (McGowan, 1973). Thus, there is no retroarticular process of the articular in *Chaohusaurus brevifemoralis*.

**Splenial.** The splenial is a thin and narrow sheet of bone that lies on the medial side of the dentary. Its complete shape is not seen in any of the specimens available, although GMPKU P-3086 shows most of the bone as previously figured (Zhou et al., 2017). The bone tapers to a point posteriorly, and probably anteriorly. The bone is longer than the dentary and seems to extend over about three quarters of the mandibular length.

**Coronoid.** The coronoid is one of the most enigmatic bones in *Chaohusaurus*. The right side of GMPKU P-3086 and the left side of AGB7403 reveal sutures between the coronoid and surangular that resemble each other closely (Figs. 5 and 6). However, this suture seems to be closed on the left side of GMPKU P-3086 and most other specimens. The coronoid occupies the area surrounding the coronoid process, mainly dorsally and medially. The bone is also visible slightly in lateral view. The bone is wide dorsally, forming a shallow basin between the coronoid process and the articular. The width of the bone is evident by comparing the lateral (Fig. 5A) and more dorsal (Fig. 5B) views of the right coronoid of GMPKU P-3086. The coronoid process is very weakly developed.

**Prearticular.** The prearticular is known from partial exposures only. The right mandibular ramus of AGB7403 is exposed laterally but medial elements have slipped ventrally relative to the lateral elements, exposing their lateral sides (Fig. 6). It is seen there that the anterior ramus of the prearticular is long and slender, tapering anteriorly to a point. Its anterior extent overlaps the most posterior part of the dentary.

#### Dentition

There are 35 teeth in the left premaxilla of the holotype, leaving about three vacant tooth positions among them. Nine teeth are recognized in the left maxilla. In total, there are 47 tooth positions in the left upper jaw ramus of the holotype. The count is similar in AGB7403, which has ten teeth in the right maxilla, and 29 teeth and about five vacant tooth positions in the left premaxilla. the tooth counts in the left maxilla and right premaxilla are uncertain. Both specimens are male individuals, so the slight difference in the total counts likely reflects individual variation. The mandibular teeth are not exposed in the holotype, but they can be counted in AGB7403, where the dentary has 42 teeth and at least eight vacant tooth positions, making the total tooth count to be about 50. This number is similar to the mandibular tooth count in *U. hataii*, which was previously estimated to be approximately 50 (Motani, 1996).

The dentition is heterodont, with slender and conical anterior teeth and robust and rounded posterior teeth. In the left maxilla of the holotype, the posteriormost six, of the nine teeth, are robust and rounded, while the anterior three appear similar to the premaxillary teeth in size and shape. The transition between the two tooth types is abrupt. The robust posterior teeth are two to three times wider in diameter than the anterior teeth. The root seems to expand basally and the pulp cavity is open, as in basal ichthyopterygians.

#### Hyoid

**Ceratohyal.** There is a pair of ceratohyal, located in-between the mandibular rami near their posterior ends. They most likely represent the first ceratohyals of reptiles (Motani et al., 2013). The ceratohyal I is a small and slender rod, which is unsuitable for suction feeding (Motani et al., 2013).

#### Vertebral column

**Atlas.** The neural arch and pleurocentrum of the atlas are exposed in the holotype (AGB7401). The neural arch is divided into the right and left halves, as in some reptiles, including ichthyosaurs (Moon & Kirton, 2016). The pleurocentrum of the atlas has a convex articular surface (Fig. 4), which is most likely the anterior surface given the preserved orientation, and that similar pleurocentra are known for *U. hataii* and *Cymbospondylus piscosus*. These bones are comparable to the corresponding axial pleurocentra in size, and are unlikely to be the intercentra. Another specimen (AGB7403) exhibits the posterior view of the pleurocentrum from a slightly lateral angle, where the posterior articular facet is concave. The bone is about only half as long as the subsequent vertebral centra (Fig. 6). There is no bone that can be positively identified as the atlantal rib in any of the specimen, although AGB7043 has a short and slender bone near the atlas, possibly representing the atlantal rib.

**Axis.** The axial neural spine is broader antero-posteriorly by about twice compared to the subsequent neural spines (Fig. 6). The neural arch has a rib facet laterally, forming the dorsal half of the synapophysis. The ventral half of the synapophysis is on the dorso-lateral part of the atlantal centrum, appearing almost triangular.

**Neural arches and spines.** The neural spines change their shapes along the vertebral column. In the cervical and anterior dorsal series, the neural spines and arches are about equal in height with each other. Both the neural spine and arch are almost perpendicular to the basal plane of the neural arch, although the axis of the former is shifted posteriorly relative to that of the latter. The neural spines gradually become longer, broader, and inclined posteriorly while the neural arches stay in approximately the same posture and height, with antero-posterior broadening in the mid-dorsal series. The lengthening of the neural spines and steepening of the posterior inclination stop early in the posterior dorsal series, and the neural spine size and angle do not change greatly in the sacral series. Then, the steepening of the neural spines restarts in the caudal series, reaching the extreme inclination, where the neural spines are almost horizontal, by about the 13th caudal vertebra. Then the inclination trend reverses, with the neural spines rapidly becoming less inclined and then start to anticline (i.e., inclined anteriorly as opposed to the normal posterior inclination) at about the 18th vertebra.

**Figure 8:**
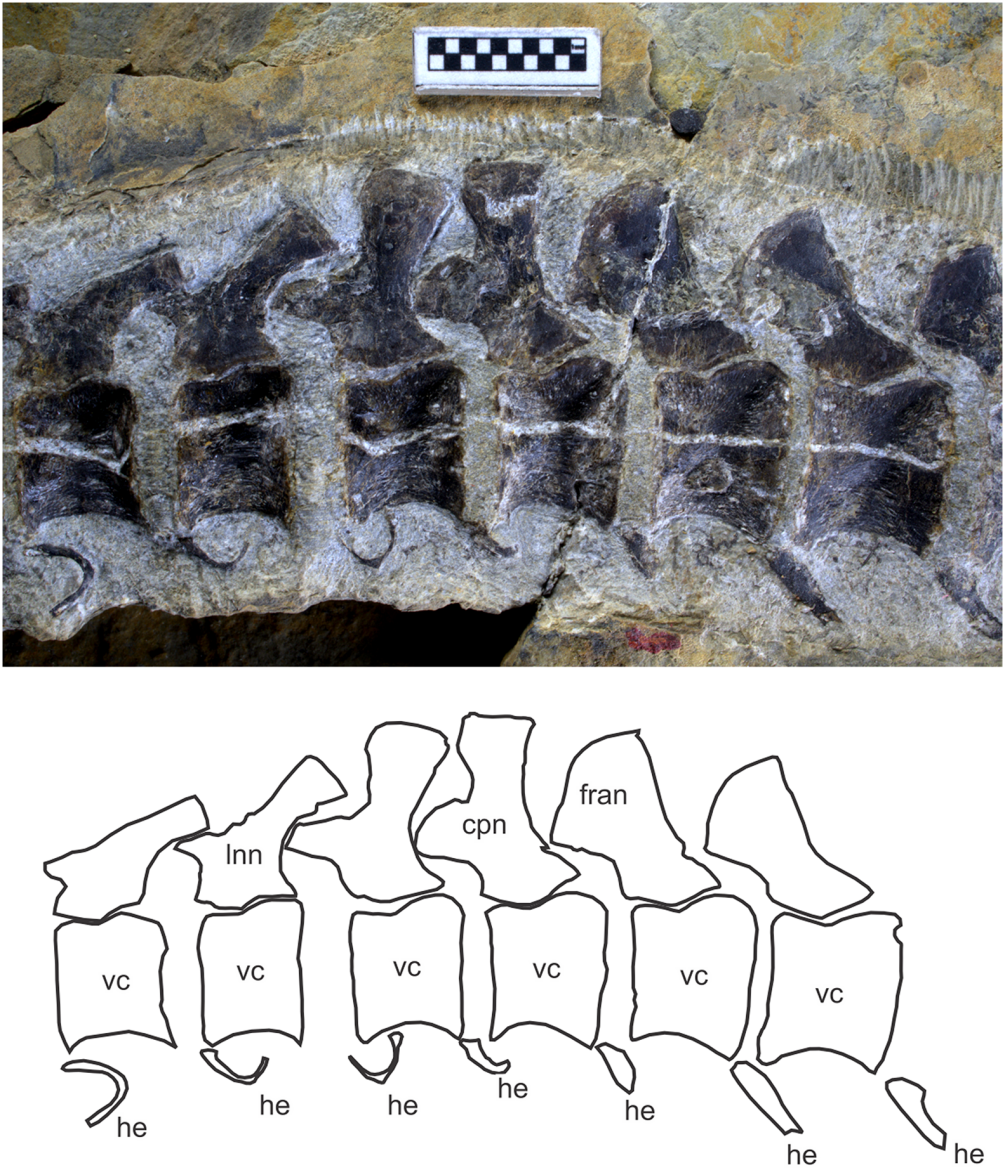
Caudal peak region of *Chaohusaurus brevifemoralis* sp. nov. in one of the paratypes (GMPKU P-3086). Scale bar is one cm.

The most notable feature of the neural spine is found in the caudal peak neural spine, which is bifurcated into two branches, one dorsal and the other antero-dorsal (cpn in Figs. 3E and 8). As noted elsewhere (Motani et al., 2018), structures that are likely homologous with these two branches also exist in *Chaohusaurus chaoxianensis* as thickened bars but the two are connected by a thin flange of bones. That flange is absent *Chaohusaurus brevifemoralis*. The caudal peak vertebra in this context was identified based on the following criteria. First, it is the second vertebra from the last narrow caudal neural spine (lnn in Figs. 3E and 8), which is inclined about 45° posteriorly. The caudal neural spines preceding this last narrow one are steeply inclined posteriorly (Fig. 8), sometimes appearing nearly horizontal. Second, the caudal peak vertebra is in front of the first caudal neural spine to have a completely rounded distal end (fran in Figs. 3E and 8), with clear anterior inclination of about 30–50°. Third, the caudal peak vertebra is usually placed where the curvature of the tail reaches its peak, although preservational bias may obscure this feature. Finally, at least in *Chaohusaurus brevifemoralis*, the caudal peak neural spine is the first anticline neural spine, although it is not necessarily the case in some specimens of *Chaohusaurus chaoxianensis*. Anticlination in this case is judged by the angle formed by two lines, one connecting the postero-dorsal corner of the neural spine with the postero-ventral corner of the neural arch, and the other connecting the antero-ventral and postero-ventral corners of the neural arch (Fig. 3E, orange lines). This angle is greater than 90° (i.e., the neural spines are inclined posteriorly, which is the norm) in most neural spines except in the posterior part of the tail, and the first anticline neural spine is where the angle first becomes less than 90° in the caudal series.

**Hemal arches and spines.** The hemal arches and spines are present starting from the third caudal vertebra to the tip of the tail. GMPKU P-3086 reveals the best representation of the hemal arches and spines in the mid-caudal region, where there is a caudal peak associated with anticlination of neural spine (see above). In front of the anticline neural spine, the hemal spines seem to be absent, while the hemal arch is U-shaped in antero-posterior view (Fig. 8; see fig. 1g of Motani et al. (2018)). The hemal arches appear absent in the caudal peak region of the holotype but this lack probably reflects a preservational bias.

**Ribs.** The first four ribs of the left side of the holotype have an expanded, fan-shaped head, which most likely represents unified capitulum and tubercle. These ribs are short and do not appear to participate in the formation of the trunk rib cage. The four pairs of ribs most likely represent cervical ribs. If so, there are five cervical vertebrae because the atlas seems to lack associated ribs.

The dorsal ribs are also single-headed, although their proximal ends typically have eight-shaped cross-section, suggesting the head is a combination of the capitulum and tubercle. Anterior and mid dorsal ribs articulate with both the neural arch and vertebral centra via a synapophysis, at least down to the 28th vertebra in the holotype. The articulation with the neural arch is lost somewhere in the posterior dorsal region, as the articular facet on the centrum moves ventrally to be completely located on the lateral side of the centrum alone. The exact location of this transition is obscure. The dorsal ribs become increasingly longer more posteriorly but they start to shorten rapidly in the last five pairs of the dorsal series, so that the last dorsal rib is only as long as the first sacral rib.

There are three sacral ribs in the holotype, identified as such based on a combination of the following observations: short; with thickened shafts; sufficiently wide at the tip to allow articulation with the ilium; oriented so that their tips converge, that is, the first one is postero-laterally inclined, the middle one laterally, and the third one antero-laterally; and located in the sacral region. The same number of sacral ribs were previously identified in AGB6253, a female specimen with embryos (Motani et al., 2014). At least the third sacral rib of the holotype seems to retain the eight-shaped cross-section of the head, seen in the more anterior ribs.

It appears that the first 10 caudal vertebrae have rib facets in the holotype, although only the first three caudal ribs are actually preserved. In AGB6253, at least the first nine caudal vertebrae have rib facets but only the first two caudal ribs are preserved. The parapophyses on the sacral and caudal centra are located at the antero-ventral corners of the centra. The rib heads are not eight-shaped in caudal ribs unlike in the more anterior ribs.

#### Shoulder girdle

**Figure 9:**
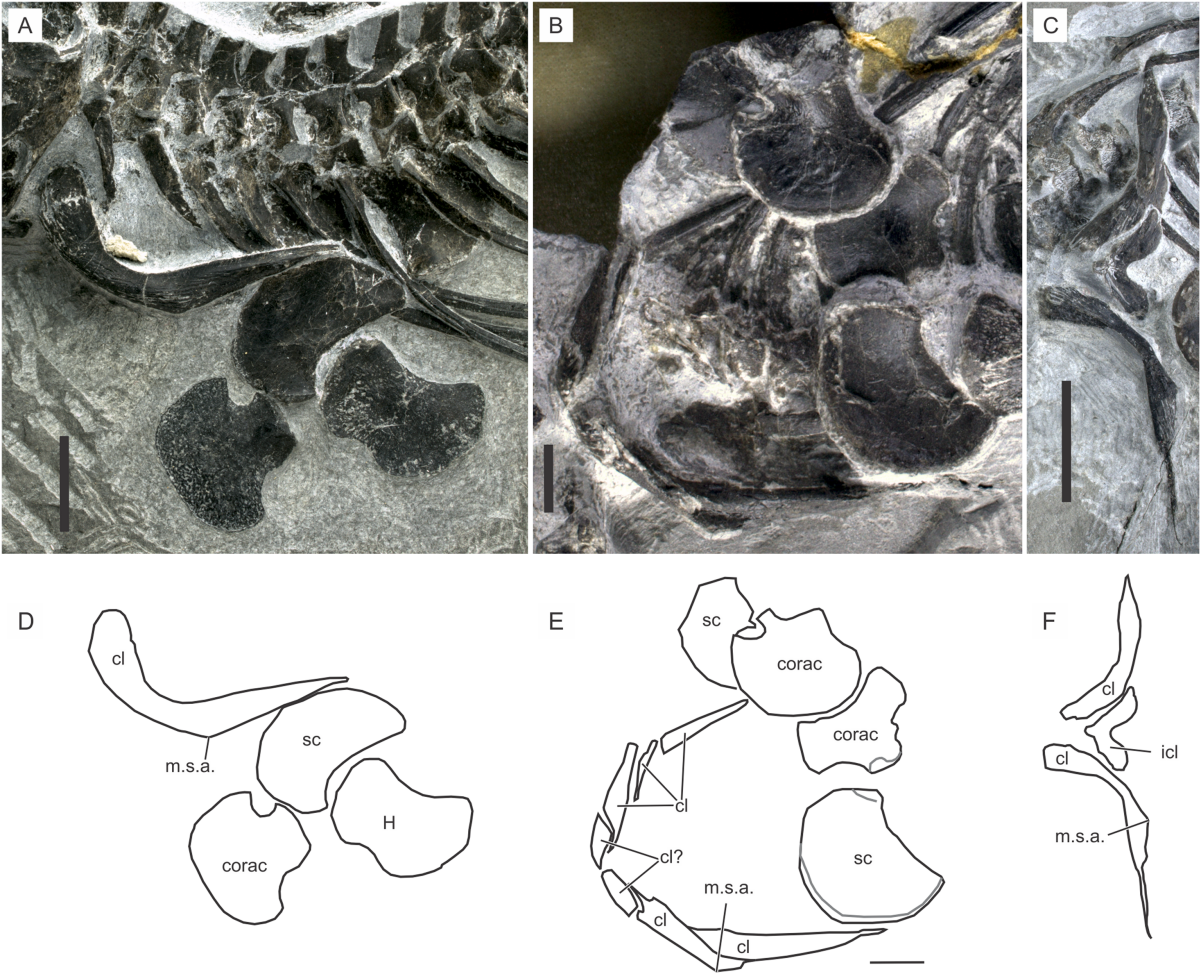
Shoulder girdle of *Chaohusaurus brevifemoralis* sp. nov. (A) AGB7405. (B) AGB6260, (C) AGB7400. (D) Approximate bone map for (A). (E) Same for (B). (F) Same for (C). See the section “Osteological abbreviations” for abbreviations. Scale bar is one cm.

**Scapula.** The scapula is a lunate plate of bone that rolls slightly to wrap around the ventro-lateral part of the shoulder rib cage, as in basal ichthyopterygians. The anterior margin is round without any indentation or emargination. The glenoid and coracoid facet forms a thickened postero-proximal corner (Fig. 9B).

**Coracoid.** The coracoid is a plate of bone that appears like a fan with a short and thickened stem (Fig. 9B). The stem ends laterally with the glenoid and scapular facets. The inter-coracoidal suture is straight in the central region only. Both the anterior and posterior margins of the coracoid have concave regions.

**Interclavicle.** The interclavicle is one of the most poorly known bones in *Chaohusaurus* in general. The complete outline of the interclavicle is seen only in two specimens that may or may not belong to *Chaohusaurus brevifemoralis*, namely AGB6607 and 7400 (see “Systematic Paleontology”). The bones in these specimens are approximately V-shaped plates (Fig. 9F), with a convex anterior margin and a concave posterior margin. The anterior process is shorter than in *Cartorhynchus*, and coarsely and strongly striated in approximately radial orientations. The overall shape of the bone would be similar to that of *U. hataii* if the posterior process of the latter interclavicle was removed. Some other specimens show in-between the two clavicles one or two pieces of bone that may appear to be a part of the interclavicle (Figs. 9B and 9C). However, after examining many specimens, these parts are most likely parts of the clavicle that wraps around the anterior side of the interclavicle and that have been cracked. It appears that the interclavicle is minimally exposed when the dermal girdle is in articulation, leading to the difficulty of seeing the bone in most specimens.

**Clavicle.** The clavicle is the most prominent bone of the shoulder girdle. It is a J-shaped bone that forms a complete U-shape when the right and left elements are articulated (Fig. 9B). AGB7400 shows a different posture of the clavicles (Fig. 9C) but this is the only specimen where the clavicle has been rotated so that its distal tip points laterally, while virtually all other specimens, including the holotype and GMPKU P-3086, have the clavicular distal tips pointing posteriorly. The condition in AGB7400 is unlikely to reflect the natural posture because the other posture is seen in over a dozen specimens.

The clavicle is thickest in two places, near the proximal end and in the midshaft region where an angle is formed antero-ventrally (Fig. 9). This midshaft angle is located in-between the coracoid and scapular articulations. The part proximal to this angle is the main body of the clavicle, which wraps around the anterior margin of the interclavicle medially and faces the gap between the coracoid and clavicle (see below) laterally. The thick proximal ends of the right and left clavicles seem to overlap slightly in front of the interclavicle, thus concealing the anterior aspect of the latter bone completely.

#### Forelimb

**Humerus.** The humerus typically has an anterior flange extending preaxially from the main shaft of the bone, as in basal ichthyosauromorphs. This flange in *Chaohusaurus brevifemoralis* is divided into a proximal and distal sub-flange as in *Chaohusaurus chaoxianensis*, but with a notable gap between the two unlike in the latter species. The relative extent of this gap changes ontogenetically, together with that of the anterior flange. In the smallest individual, the sub-flanges appear virtually absent (Fig. 9A), with the anterior margin of the humerus being concave. The concave margin has radial, rather than longitudinal, striations, suggesting the presence of a very narrow anterior flange along the margin. The sub-flanges appear proximally and distally and extend toward the center of the anterior margin with growth. The gap between them therefore narrows with growth, although it never disappears completely. In *Chaohusaurus chaoxianensis*, a notch-like incision exists between the two sub-flanges, instead of an obvious gap as in *Chaohusaurus brevifemoralis*.

**Figure 10:**
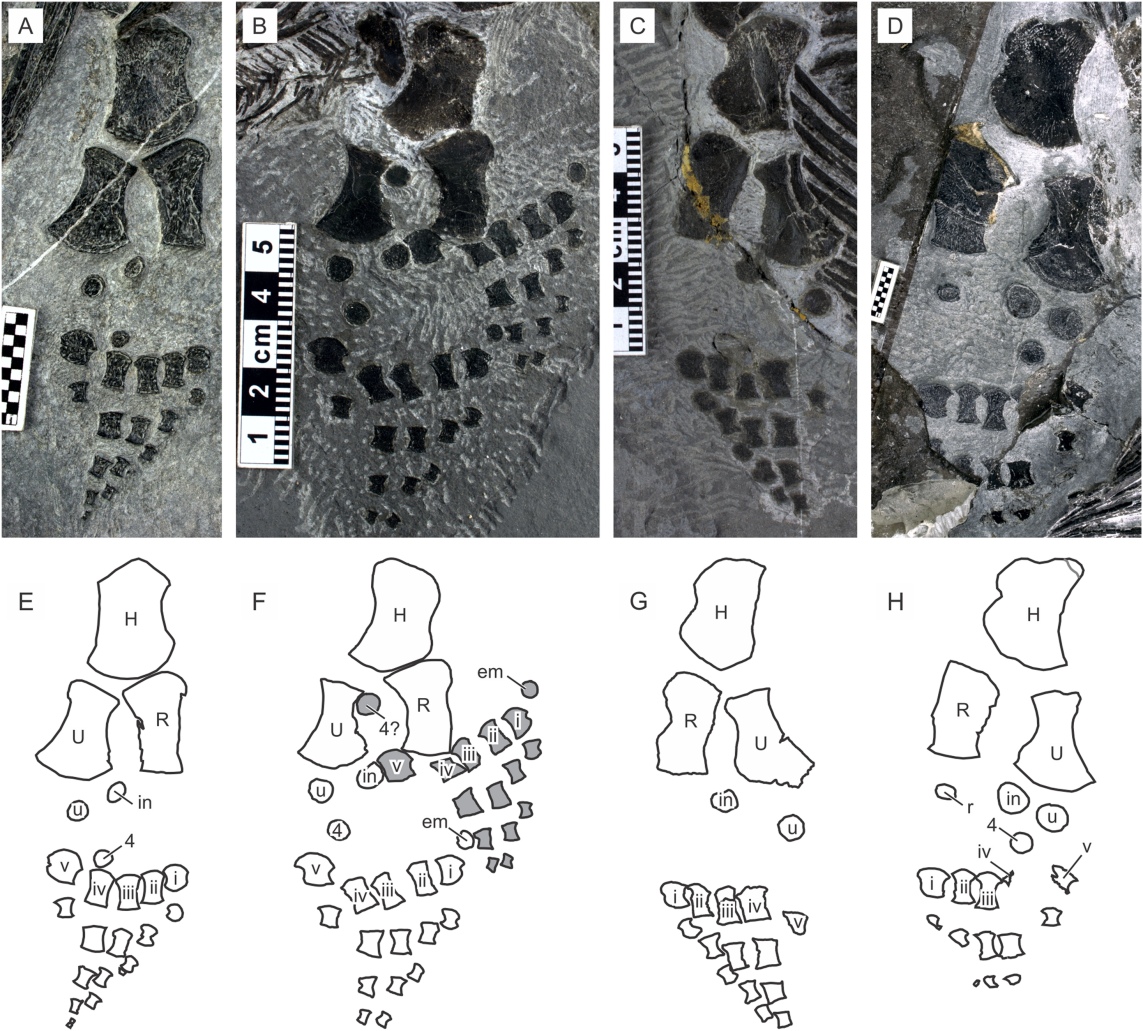
Forelimb of *Chaohusaurus brevifemoralis* sp. nov. (A) AGB7405. (B) AGB6260, (C) AGB7400. (D) AGB7408. (E) Approximate bone map for (A). (F) Same for (B). (G) same for (C). (H) Same for (D). See the section “Osteological abbreviations” for abbreviations. Scale bar is one cm.

The head of the humerus is located postero-proximally. Its ossification is usually incomplete, with only the largest specimen revealing a humeral head that is partly ossified (Fig. 10D). The degree of ossification of the humeral head is used to judge the relative degree of osteological maturity in thunnosaurian ichthyosaurs (Johnson, 1977). Similar usage is difficult in *Chaohusaurus brevifemoralis* because of the poor ossification in almost all specimens (Fig. 10).

**Radius.** The radius is a flattened long bone that is wider proximally than distally. The peripheral shaft of the bone seems to be absent because the entire anterior margin of the bone has radial orientation of striation that are perpendicular to the margin, rather than those that are parallel to the main axis of the bone as in the proper shaft region. As a result, there is a band of “anterior flange” extending along the entire peripheral margin of the bone, making the bone appear wide. The anterior flange widens proximally, where there is the antero-proximal flange of the radius in *Chaohusaurus chaoxianensis*. In *Chaohusaurus chaoxianensis*, the contrast in widths between this antero-proximal flange and the more distal part of the flange is conspicuous, with an abrupt change. However, in the present species, the difference is much smaller because the flange gradually widens proximally without any abrupt change. As a result, the antero-proximal flange of the radius appears small at the first sight.

**Ulna.** The ulna is an elongated and flattened bone with a proximal shaft and a distal fan, as in basal ichthyopterygians. The distal fan of the ulna typically appears asymmetric relative to the longitudinal axis of the bone, developing more postaxially than preaxially. This morphology differs from that in a typical ulna of *Chaohusaurus chaoxianensis* where the fan is more symmetrical except in one individual where one of the ulnae has a weakly asymmetrical distal fan (AGB6259; compare Motani et al. (2015a: fig. 1C) and Motani et al. (2015b: figs. 2E, F)).

**Carpals.** Much of the carpus region is without hard tissue. There are three disk-shaped carpal ossifications, corresponding to the intermedium, ulnare, and fourth distal carpal, in nearly all individuals regardless of body size. Exceptions are a large specimen which seems to have only two carpal bones (AGB6260; Fig. 10C), the terminal embryo described elsewhere that lacks any carpal ossification (AGB6253), and the largest specimen with four carpals, adding the radiale to the list above of three bones (AGB7408). The largest individual of *Chaohusaurus chaoxianensis* also has four carpals (Motani et al., 2015c), but this individual is smaller than large specimens of *Chaohusaurus brevifemoralis* with only three carpals. Carpal ossifications in *Chaohusaurus brevifemoralis* appear smaller than those of *Chaohusaurus chaoxianensis* relative to the carpus length.

**Metacarpals.** There are five metacarpals in all specimens, except in one specimen (AGB6258) where six metacarpals are present in both right and left forelimbs (Fig. 10B). The extra metacarpal in this exceptional individual is located preaxially, as in some hupehsuchian ichthyosauromorphs. Unlike in hupehsuchians, however, there is no ossification for an extra preaxial distal carpal that would form the base of the extra metacarpal. Also, there are no phalanges that extends distally from this extra metacarpal. The first and fifth distal carpals lack a complete shaft along their peripheral margins, which are notched. These bones are not completely lunate as in most basal ichthyopterygians because of the notch.

**Manual phalanges.** The forelimb is hypophalangeal, with a phalangeal formula is 1-3-3-3-1. The third phalanx of the second digit is not always present, making the formula 1-2-3-3-1 in some specimens. The phalangeal formula does not seem to depend on size. As in basal ichthyopterygians, most phalanges are flattened cylinders that are approximately symmetrical across the long axis but elements along the peripheral margin of the limb tend to be asymmetrical. The phalanges are well spaced from each other, more so than in *Chaohusaurus chaoxianensis*.

#### Pelvic girdle

**Figure 11:**
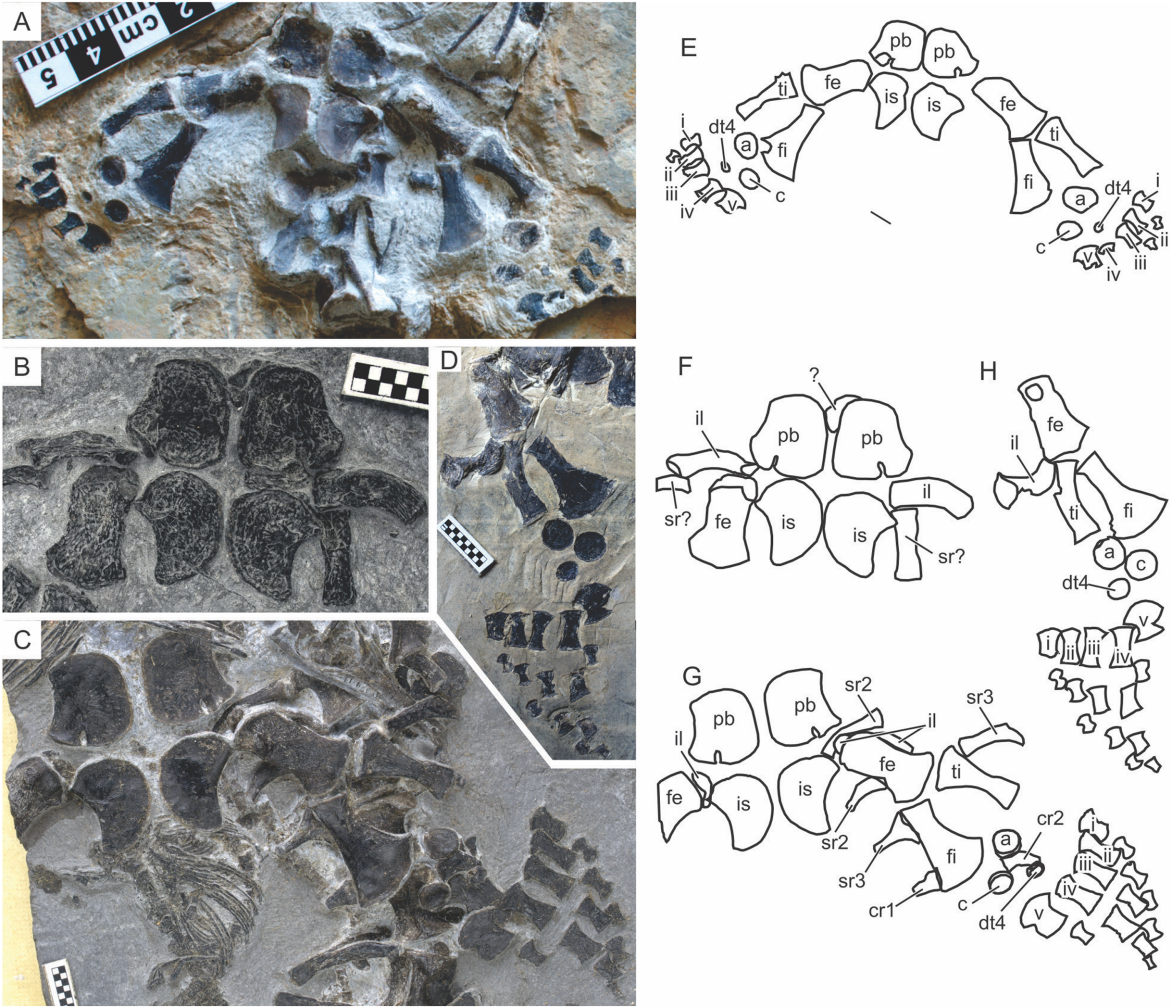
Pelvic girdle and hind limb of *Chaohusaurus brevifemoralis* sp. nov. (A) GMPKU P-3086 (one of the paratypes). (B) AGB5846b. (C) AGB6253. (D) AGB7402. (E) Approximate bone map for (A). (F) Same for (B). (G) Same for (C). (H) Same for (D). See the section “Osteological abbreviations” for abbreviations. Scale bars are made of one mm squares.

**Ilium.** The ilium changes its shape with growth. In juveniles, it is essentially a short and flattened rod of even thickness that curves slightly (Fig. 11A). In adults, the curvature becomes steeper and a conspicuous iliac blade is formed in the dorsal side of distal part, which is approximately horizontal in natural posture (Fig. 11C). The acetabular facet also expands, resulting in a constriction of the bone between the acetabulum and the iliac shaft.

**Pubis.** The pubis is the largest of the pelvic bones, although it is not remarkably larger than the ischium (Figs. 11A and 11B). The bone is thickened along its antero-lateral margin, toward the acetabular facet that is located at the posterior end of this margin. The acetabular facet is robust and faces postero-laterally. There is an obturator foramen that opens posterolaterally, just medial to the acetabular fact (Figs. 11A and 11B). Because of this location, the medial margin of the foramen is thick while the lateral margin is much thinner. The inter-pubic margin is approximately straight, although the right and left elements are usually preserved at a distance from each other.

**Ischium.** The ischium is a lunate bone (Fig. 11). Its antero-lateral corner is thickened to form the acetabular facet, which is thinner than the corresponding facets in the pubis and ilium. The facet is also narrower than the corresponding facet of the pubis. As a result, the acetabulum is not oriented completely laterally but slightly posteriorly (Fig. 11B).

#### Hind limb

**Femur.** The femur is a robust bone that is wider distally than proximally, with a weakly constricted shaft in-between. The shaft in many specimens may appear wider than in life depending on the degree of compression during preservation. There is a trochanter on the ventral side of the bone, located slightly distal to the head. Its homology with the known ventral femoral trochanters of reptiles, such as the internal trochanter and fourth trochanter (Romer, 1956), is unknown. There is no fossa on the femoral sides. The distal condyle of the femur is flattened, making the knee joint inflexible.

**Tibia.** The tibia is a cylindrical bone in the holotype, unlike other limb elements that are flattened at least to some extent. In most other specimens, however, the bone has been compacted to be a flat element. The tibia is wider proximally than distally, and slightly curved posteriorly toward the distal end.

**Fibula.** The fibula is a flattened long bone with a fan-shaped distal end. The fan develops anteriorly to the shaft, curving the anterior margin of the shaft more strongly than the posterior margin, which in turn is almost straight. This relationship between the shaft and the fan is not obvious in most specimens because of compaction, but is seen at least in better-preserved specimens, including the holotype and AGB6253.

**Tarsals.** Much of the tarsal region is without hard tissue. There are three tarsal ossifications of various sizes, representing the calcaneum, astragalus, and the fourth distal tarsal. This number seems to be constant regardless of body size. They are all disk-shaped and without three-dimensional sculpture. The astragalus is usually the largest, and the fourth distal tarsal the smallest, although the relative size between the astragalus and calcaneum is highly variable depending on the individual.

**Metatarsals.** The metatarsals are similar to the metacarpals in general shape, although the former have shafts that are more slender than the latter, at least in the holotype. This difference is not the result of a preservational bias because the left metacarpals and metatarsals are preserved next to each other in the specimen (Fig. 2).

**Pedal phalanges.** The pedal phalangeal formula is 1-2-3-3-1 where countable. As with the metatarsals, the pedal phalanges are more slender than corresponding manual phalanges.

### Phylogenetic analysis

**Figure 12:**
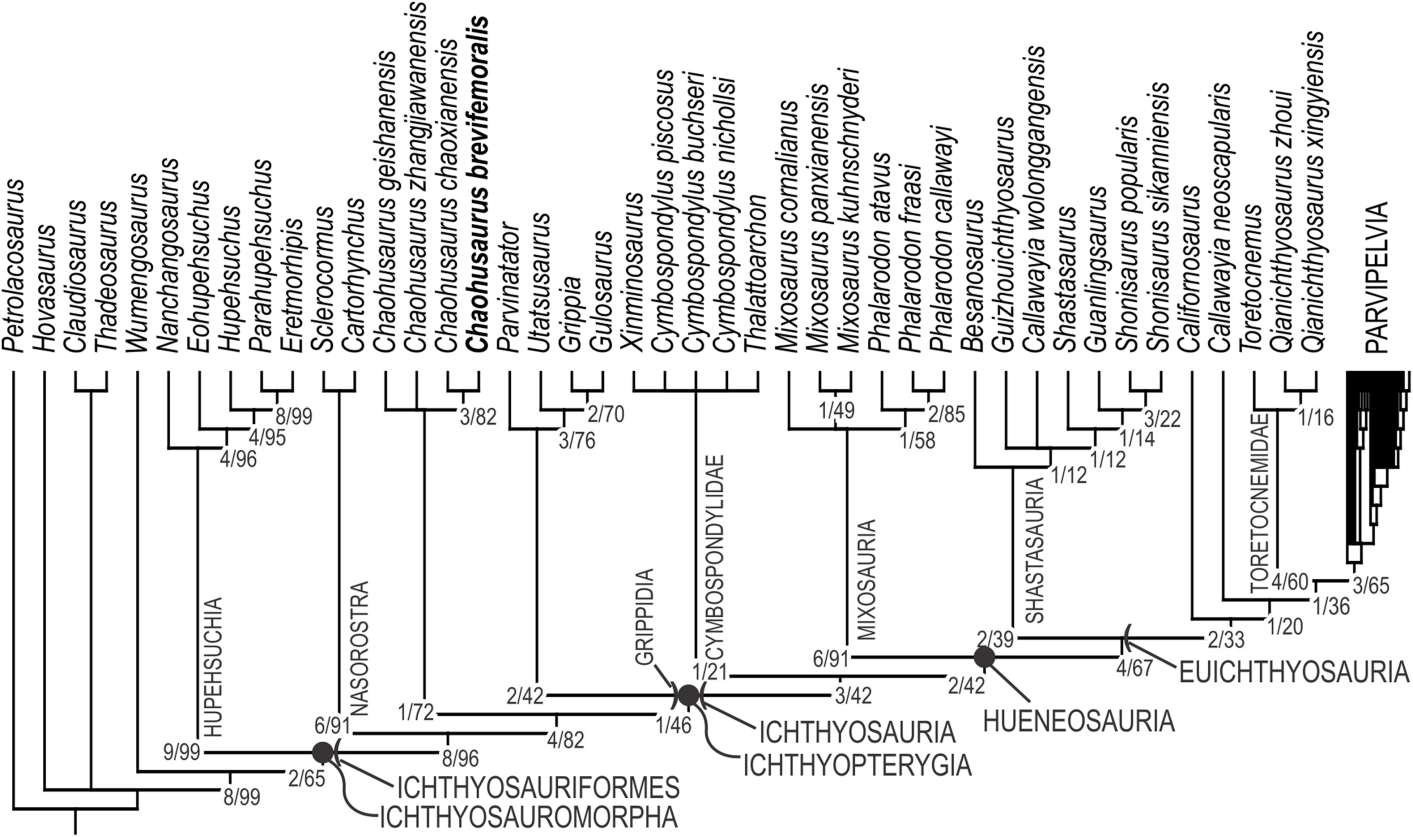
Phylogenetic hypothesis of basal Ichthyosauromorpha including *Chaohusaurus brevifemoralis* sp. nov. Numbers given to nodes are Bremer support value/bootstrap value. See text for how they were computed.

Both software packages yielded an identical length of 713 steps for the shortest trees (CI = 0.363, RI = 0.787). TNT found more than 10,000 equally parsimonious trees, the strict consensus trees of which is given in Fig. 12, with Bremer support and bootstrap values. We tried removing *Wumengosaurus* from the outgroup, but it did not affect the consensus topologies. *Chaohusaurus brevifemoralis* appeared as the sister taxon of the sympatric species, *Chaohusaurus chaoxianensis*. This clade has a Bremer support value of 3, and bootstrap support of 82%. The clade is supported by the following unambiguous apomorphies: 98(1), 116(1), 118(1) and 135(1). In contrast, the genus *Chaohusaurus* is weakly supported, without any unambiguous apomorphies—detailed studies of *Chaohusaurus geishanensis* and *Chaohusaurus zhangjiawanensis* are necessary to clarify the nature of the clade.

The purpose of the present phylogenetic analysis is to locate the new species in the phylogenetic tree of Ichthyosauromorpha, and not to revise the phylogeny of the group. However, it is still worth describing the broader tree briefly. The strict consensus tree topology did not differ drastically from what were published before based on the relevant series of data matrix (Motani et al., 2015a, 2017; Jiang et al., 2016; Ji et al., 2016). The interrelationships among major clades within Ichthyosauromorpha remained the same throughout these studies. Relationships within some of the major clades have changed through the studies but the present result has minimal difference from the tree given in the last iteration of the series (Motani et al., 2017), with the only difference found in the breakdown of the clade for the genus *Mixosaurus*. As a result, the distribution of apomorphies along the tree remained similar to what was found in the previous iteration. For example, Ichthyosauriformes is supported by the following character state changes: 11(1), 36(1), 59(1), 100(1), 115(1), 123(1), 144(1), 178(1) and 198(1). Ichthyopterygia shares the following unambiguous apomorphies: 48(1), 89(1), 95(2), 99(0) and 140(1), while Grippidia shares the following two: 34(1) and 113(0).

## Discussion

The holotype of *Chaohusaurus brevifemoralis* sp. nov (AGB7401) revealed for the first time the three-dimensional relationship between the orbit and the scleral ring in basal Ichthyosauromorpha. It has been known that the skull roof was constricted in the middle of the orbit in basal ichthyosauriforms (Mazin, 1981; Motani, Minoura & Ando, 1998; Cuthbertson, Russell & Anderson, 2013b) and hupehsuchians (Chen et al., 2014a, 2014c) but the ramification of this morphology on the eyeball was never clear because the skulls are usually flattened through postmortem compaction. The scleral rings of the holotype retain their three-dimensional depths (Fig. 4). Both of them are exposed dorsally, through the embayment of the skull roof formed by the medial excursion of the dorsal orbital margin, in addition to the exposure through the main part of the orbit. The dorsal exposure reveals the peripheral wall of the scleral ring while the orbital exposure shows the central frustum wall. The function of this dorsal embayment is unclear, while there must be a disadvantage from the additional exposure from the dorsal aspect making the eyeball vulnerable to stresses from that direction. It is unlikely that the embayment allowed the animal to look up better—the limited space inside the eye socket may not have allowed much movement of the eyeballs, even though it appears that looking up to some extent was a part of the lifestyle of *Chaohusaurus brevifemoralis*, with the main part of the orbit facing not completely laterally but slightly dorsally. Despite the ambiguity of function, this embayment and the consequent dorsal exposure of the eyeball was a wide-spread feature found in virtually all ichthyosauromorphs outside of Ichthyosauria.

The anterior flange of the humerus of *Chaohusaurus* was considered to be notched until recently, based on *Chaohusaurus chaoxianensis* that was the only species in which the flange was completely preserved (Motani & You, 1998a, 1998b). However, a new picture has now emerged based on recent findings of new specimens and taxa, including the present species. The margin is indeed concave in three of the four *Chaohusaurus* species, namely *Chaohusaurus geishanensis*, *Chaohusaurus zhangjiawanensis* and *Chaohusaurus brevifemoralis*, although there is a narrow band of flange at least in the latter two species based on the radial orientations of the surface striations perpendicular to the anterior margin. The concave flange morphology is most likely a basal character in Ichthyosauromorpha because it is also present in *Cartorhynchus lenticarpus* (Motani et al., 2015a) and hupehsuchians (Chen et al., 2014a, 2014b, 2014c; Wu et al., 2016). Therefore, the notched morphology in *Chaohusaurus chaoxianensis* is an autapomorphy of the species. In the light of this recognition, the complete and convex humeral anterior flanges in grippidians (*Utatsusaurus*, *Grippia* and *Gulosaurus*) (Motani, 1997, 1998; Cuthbertson, Russell & Anderson, 2013a), represent a shared apomorphy with the rest of the basal ichthyopterygians.

The position of the pineal foramen relative to the orbit has been used as a character with phylogenetic information content (reproduced as Character 59 in the present study). Typically, the pineal foramen is posterior to the orbit in basal forms and located in-between the orbits in derived forms. *Chaohusaurus brevifemoralis* represents an intermediate state. In specimens with dorso-ventral preservational compaction, the pineal foramen tends to be mostly posterior to the line connecting the posterior limits of the orbits. However, the anterior end of the foramen very slightly crosses the line. More problematically, this positioning varies with specimens. In specimens with oblique preservational compaction, the foramen is often found to be largely in-between the orbits. The same is true in other species of the genus *Chaohusaurus*.

Participation of the frontal in the orbital rim has been considered an important basal feature of ichthyosauriforms. It is minimally present in *Grippia longirostris* and *U. hataii* because the pre- and postfrontals leave only a narrow gap between them to allow the frontal to be a part of the orbital margin, unlike in *Cartorhynchus lenticarpus* where the frontal forms a substantial portion of the orbital rim as in basal neodiapsids. In *Chaohusaurus brevifemoralis*, this participation is absent because of a narrow contact between the pre- and postfrontals that excludes the frontal from the orbital rim. Despite this difference, it is important to recognize that the overall morphology of the orbital margin in *Grippia* and *Utatsusaurus* is much closer to that of *Chaohusaurus brevifemoralis* than to the same of *Cartorhynchus*. That is, the dorsal part of the orbital rim has a medial excursion corresponding to the narrow lateral embayment of the skull roof, exposing the eyeball dorsally. The only difference is whether the pre- and postfrontals contact each other narrowly or not at the deepest part of the embayment. A similar situation is known in hupehsuchians, where some specimens are preserved with pre- and postfrontals in contact while others have a narrow gap between the two, exposing the frontal to the orbital margin. Therefore, the narrow contact between the pre- and postfrontal may not be a significant phylogenetic character. Based on the tree topology in Fig. 12, the contact in *Chaohusaurus* is not necessarily homologous with the robust articulation between the pre- and postfrontals in Ichthyosauria.

Participation of the frontal in the margin of the upper temporal fenestra was once suggested in *U. hataii* (Motani, Minoura & Ando, 1998) but a later study questioned this morphology (Cuthbertson, Russell & Anderson, 2013b). Some of the sutures in the specimen in question were not completely clear, leaving room for controversy. Based on well-preserved specimens with unambiguous cranial sutures at hand, it is clear that at least in *Chaohusaurus brevifemoralis* the frontal entered the anterior margin of the upper temporal fenestra, in a manner similar to what was previously described for *U. hataii*. This morphology is unusual among diapsids but it is necessary to recognize that it existed in basal ichthyosauriforms. Reevaluation of the feature in other basal ichthyosauriforms is needed based on well-preserved specimens.

The squamosal is the most problematic bone in the cranium that warrants a discussion. The bone is interpreted here to be located in-between the supratemporal and quadratojugal, near the center of the height of the cheek, without participating in the upper temporal fenestra. This interpretation differs from a previous one that located the bone along the dorsal margin of the cheek that forms the lateral margin of the upper temporal fenestra. This interpretation was based on two specimens (GMPKU P-3086 and P-3101), but the same study located the squamosal in the same position as in the present work in another specimen (GMPKU P-3188) (Zhou et al., 2017). We reinterpreted GMPKU P-3086 and concluded that the squamosal did not reach the upper temporal fenestra. The apparent contact between the bone and the fenestra is an artifact of damages in the lateral margin of the upper temporal fenestra that expanded the opening laterally, and disarticulation and translation of the bones in the area. If the preserved morphology is original and the squamosal was located along the upper temporal fenestra: (1) the height of the lower temporal fenestra would become about three-quarters of the total cheek height when the proportion is only about half in other specimens; (2) the lateral margin of the upper temporal fenestra would be located too laterally relative to the parietal—the supratemporal process of the parietal extends about halfway through the width of the upper temporal fenestra in other specimens when it is only about a third in the specimen in question; (3) similarly, the upper temporal fenestra would be too large relative to the orbit—the fenestra is less than half of the orbit in maximum diameter in other specimens but the proportion is much larger than half in the specimen in question (Table 1); and (4) the preserved margin of the upper-temporal fenestra would need to be bent unnaturally in three-dimension to reach the squamosal. The problems would disappear if the actual lateral margin of the upper temporal fenestra was more dorsally located than the squamosal, and breakage of the margin led to expansion of the upper temporal fenestra while displacing the margin ventrally. However, scrutiny using 3D reconstruction of CT images would be necessary in the future to reach a solid conclusion.

It would be useful to compare the morphology of *Chaohusaurus* spp. with those of grippidians (*Utatsusaurus*, *Grippia*, *Gulosaurus*, and *Parvinatator*), from the Lower Triassic. However, detailed comparisons are difficult given the quality of the specimens available for grippidian taxa, and the lack of understanding of the morphology of *Chaohusaurus geishanensis* and *Chaohusaurus zhangjiawanensis*. as well as the lack of three-dimensional reconstruction of the skull in all of the relevant specimens. We therefore consider it premature to present a detailed comparison. However, it is useful to point out some of the unambiguous character state changes. For example, one of the most remarkable differences between grippidians and the more basal ichthyosauriforms is the clear presence of the lower temporal fenestra in the latter as a narrow but deep ventral incision between the postorbital and quadratojugal, while it is lost in the former. Also, the interpterygoid vacuity is open in *Chaohusaurus* and other basal ichthyosauriforms but closed in grippidians and some other ichthyopterygians. Postcranially, *Chaohusaurus* and other basal ichthyosauriforms all lack the pisiform and exhibit delayed ossification of the mesopodium while grippidians and some other ichthyopterygians possess the bone and lack any sign of delay in mesopodial ossification. Additionally, as discussed above, humeral anterior flange morphology differs between the two assemblages.

The anterior end of the clavicle is almost always preserved in the neck region (Fig. 8), and there is a gap between the clavicle and coracoid in most specimens. Given its occurrence in most of the specimens, this gap probably reflects the anatomical positions of the bones in life. The presence of such a gap is unusual among reptiles but a similar gap was identified in *Ophthalmosaurus* (Moon & Kirton, 2016). Note, however, that the gap is absent in another thunnosaurian ichthyosaur, *Stenopterygius* (Johnson, 1979), so it is difficult to establish homology between the gaps in *Chaohusaurus brevifemoralis* and *Ophthalmosaurus* without further studies of this character. Another possible interpretation of the preserved gap in *Chaohusaurus brevifemoralis* is that there was a shared mechanism to anteriorly displace the clavicles postmortem, but the hypothesis currently lacks evidence and a candidate mechanism.

The co-existence of three species of *Chaohusaurus* in a small geographic region may sound strange. However, as we argued elsewhere, only two of the three, namely *Chaohusaurus chaoxianensis* and *Chaohusaurus brevifemoralis*, are truly sympatric (Motani et al., 2018). The two species differ in average body size (Motani et al., 2018), with *Chaohusaurus brevifemoralis* being larger than *Chaohusaurus chaoxianensis*. It is therefore likely that the two utilized different resources in the same geographic region. Phylogenetic analyses suggested a sister-group relationship between the two species. It may be hypothesized that the speciation between the two species involved pursuit of different recourses, at least partly facilitated by the evolution of different body sizes.

## Conclusions

The new ichthyosauriform *Chaohusaurus brevifemoralis* is distinguished from existing species based on a suite of features, including the bifurcation of the neural spines near the caudal peak and short femora relative to the body. These differences are unlikely to be sexual dimorphisms, which are present in the species as an independent suite of characters (Motani et al., 2018). *Chaohusaurus brevifemoralis* is larger than its sister taxon, *Chaohusaurus chaoxianensis*, in body size. The two species were sympatric but likely pursued different resources from each other’s.

## Supplemental Information

10.7717/peerj.7561/supp-1 Supplemental Information 1 Taxon-character data matrix of Ichthyosauromorpha. Save the file as a NEXUS file.

10.7717/peerj.7561/supp-2 Supplemental Information 2 Character number correspondences between the present study and Motani et al. (2017).

## Osteological abbreviations

a: astragalus
an: angular
ar: articular
atn: atlantal neural spine
atp: atlantal pleurocentrum
axc: axis centrum
axn: axis neural spine
axr: axis rib
bo: basioccipital
bs: basisphenoid
c: calcaneum
c3: 3rd centrum
ch: ceratohyal
cl: clavicle
co: coronoid
corac: coracoid
cpn: caudal peak neural spine
cr: caudal rib
d: dentary
d.v.f.: descending ventral flange of parietal
em: extra preaxial metacarpal
eo: exoccipital
f: frontal
fe: femur
fi: fibula
fran: first round anticlined neural spine
H: humerus
he: hemal arch
icl: interclavicle
il: ilium
in: intermedium
is: ischium
j: jugal
l: lacrimal
lnn: last narrow neural spine
m: maxilla
n: nasal
n3-5: 3rd to 5th neural spines
pb: pubis
pl: palatine
po: postorbital
pra: prearticular
prf: prefrontal
ps: parasphenoid
pt: pterygoid
ptf: postfrontal
p.sl.: parietal slope
q: quadrate
qj: quadratojugal
R: radius
r: radiale
sa: surangular
sc: scapula
sp: splenial
sq: squamosal
sr: sacral rib
st: supratemporal
ti: tibia
U: ulna
u: ulnare
v10-v70: vertebrae
vc: vertebral centrum
i-v: metacarpal or metatarsal
4: fourth distal carpal.

## Institutional abbreviations

AGM: Anhui Geological Museum, Hefei, Anhui, China
GMPKU: Geological Museum, Peking University, Beijing, China
IVPP: Institute of Vertebrate Paleontology and Paleanthropology, Chinese Academy of Sciences, Beijing, China
NGM: Nanjing Geological Museum.

## Additional Information and Declarations

### Data Availability

The following information was supplied regarding data availability: Raw data is available in the Supplemental Files.

### New Species Registration

The following information was supplied regarding the registration of a newly described species: Publication LSID: urn:lsid:zoobank.org:pub:3FA09089-C940-4499-ABBC-B48F0F70F38E *Chaohusaurus brevifemoralis* LSID: urn:lsid:zoobank.org:act:B0085E53-C6E4-4C2E-96FF-6D485D3585BA.
