# Supplementary material for: The new ichthyosauriform Chaohusaurus brevifemoralis (Reptilia, Ichthyosauromorpha) from Majiashan, Chaohu, Anhui Province, China
Source: PeerJ. 2019 Sep 9;7:e7561. doi: 10.7717/peerj.7561 (PMC6741286; doi:10.7717/peerj.7561)
Supplement: Supplemental Information 2 [file peerj-07-7561-s002.docx]

**Supplementary Table S1.**

Character number correspondences between the present study and Motani et al. (2017). Note that there was a rearrangement of character order between the text and nexus file in Motani et al. (2017) due to version differences but the content of the matrix remained constant.

| Character name | Present study | Motani et al. (2017) | Motani et al. (2017) Nexus File |
| --- | --- | --- | --- |
| Premaxilla dorsal process | 1 | 1 | 1 |
| Premaxilla ventral process | 2 | 2 | 2 |
| Maxilla anterior process | 3 | 3 | 3 |
| Maxilla dorsal lamina | 4 | 4 | 4 |
| Maxilla prefrontal contact | 5 | 5 | 5 |
| Maxilla external naris contact | 6 | 6 | 6 |
| Maxilla longer than premaxilla | 7 | 52 | 7 |
| External naris orientation | 8 | 7 | 8 |
| Shallow groove anterior to the exn | 9 | 8 | 9 |
| Narial shelf | 10 | 9 | 10 |
| Nasal anteriorly extending beyond exn | 11 | 10 | 11 |
| Nasal parietal contact lateral to frontal | 12 | 11 | 12 |
| Nasal postfrontal contact | 13 | 12 | 13 |
| Descending process of the nasal on the dorsal border of the nares | 14 | 13 | 14 |
| Nasals rostrally reaching snout tip | 15 | 58 | 15 |
| Processus narialis of prefrontal | 16 | 14 | 17 |
| Supraorbital crest on prefrontal and postfrontal | 17 | 15 | 20 |
| Prefrontal-postfrontal contact | 18 | 16 | 18 |
| Prefrontal exposure in UTF | 19 | 53 | 19 |
| Postfrontal medial extension | 20 | 18 | 25 |
| Supratemporal antero-medial extension | 21 | 19 | 36 |
| Supratemporal-postorbital contact | 22 | 20 | 37 |
| Supertemporal / squamosal relative size | 23 | 56 | 38 |
| Sagittal eminence | 24 | 21 | 39 |
| Frontal dorsal exposure | 25 | 22 | 26 |
| Frontal participation in utf | 26 | 23 | 27 |
| Squamosal triangular shape | 27 | 24 | 33 |
| Squamosal participation in utf | 28 | 25 | 34 |
| Squamosal-quadrate articulation | 29 | 55 | 35 |
| Postorbital postero-dorsal corner | 30 | 26 | 23 |
| Postorbital participation in utf | 31 | 27 | 24 |
| Jugal anterior margin | 32 | 28 | 21 |
| Jugal/quadratojugal lateral contact | 33 | 29 | 22 |
| Lower temporal arch between jugal and quadratojugal | 34 | 30 | 40 |
| Quadratojugal | 35 | 31 | 41 |
| Quadratojugal exposure | 36 | 32 | 42 |
| Parietal ridge | 37 | 33 | 28 |
| Parietal supratemporal process | 38 | 34 | 29 |
| Parietal anterior processes | 39 | 35 | 30 |
| Parietal-frontal suture inter-digitation | 40 | 54 | 31 |
| Anterior terrace of utf | 41 | 36 | 43 |
| Basioccipital peg | 42 | 37 | 44 |
| Basioccipital extracondylar area | 43 | 38 | 45 |
| Basioccipital/atlas articulation convexity | 44 | 39 | 46 |
| Ventral notch in the extracondylar area of the basioccipital | 45 | 40 | 47 |
| Pterygoid, transverse flange | 46 | 41 | 48 |
| Basipterygoid processes | 47 | 42 | 49 |
| Interpterygoid vacuity | 48 | 43 | 50 |
| Ectopterygoid | 49 | 44 | 51 |
| Shape of the paroccipital process of the opisthotic | 50 | 45 | 52 |
| Stapes proximal head | 51 | 46 | 53 |
| Cheek orientation | 52 | 47 | 54 |
| Overbite | 53 | 48 | 55 |
| Prenarial snout longer than the postorbital skull | 54 | 51 | 59 |
| Snout extremely slender | 55 | 49 | 56 |
| Snout, constriction | 56 | 50 | 57 |
| Snout flattened | 57 | 60 | 58 |
| Scleral ring extensively ossified, filling or almost filling the orbit | 58 | 57 | 60 |
| Pineal foramen posterior to or between orbits | 59 | 59 | 32 |
| Anterior orbital margin | 60 | 17 | 16 |
| Dorsal orbital margin | 61 (new) | *—* | *—* |
| Angular lateral exposure at its maximum depth | 62 | 61 | 61 |
| Coronoid region | 63 | 62 | 62 |
| Root striations | 64 | 63 | 63 |
| Plicidentine | 65 | 64 | 64 |
| Bony fixation of teeth | 66 | 65 | 65 |
| Tooth horizontal section | 67 | 66 | 66 |
| Tooth size relative to the skull width | 68 | 67 | 67 |
| Dental groove | 69 | 69 | 69 |
| Anterior sockets | 70 | 70 | 70 |
| Maxilla multiple tooth row | 71 | 71 | 71 |
| Dentary labial shelf | 72 | 72 | 72 |
| Posterior tooth crown | 73 | 73 | 73 |
| Tooth crown surface of at least one maxillary tooth with mesiodistal ridge | 74 | 74 | 74 |
| Premaxillary teeth presence | 75 (new) | (68) | (68) |
| Maxillary teeth precence | 76 (new) | (68) | (68) |
| Ossified sternum | 77 | 75 | 75 |
| Ossified cleithrum | 78 | 76 | 76 |
| Clavicle orientation at proximal end | 79 | 77 | 77 |
| Clavicle scapular process length distal to clavicular main body | 80 | 78 | 78 |
| Interclavicle anterior process separating clavicles | 81 | 79 | 79 |
| Interclavicle posterior process | 82 | 80 | 80 |
| Scapular blade shaft | 83 | 81 | 81 |
| Scapula anterior flange | 84 | 82 | 82 |
| Scapula antero-proximal extension toward clavicle | 85 | 83 | 83 |
| Prominent acromion process of scapula | 86 | 84 | 84 |
| Scapula posterior extension | 87 | 85 | 85 |
| Scapular axis and glenoid facet orientations | 88 | 86 | 86 |
| Coracoid facet on scapula | 89 | 87 | 87 |
| Coracoid parasagittal length vs. transverse width | 90 | 88 | 88 |
| Coracoid foramen | 91 | 89 | 89 |
| Coracoid anterior notch or concavity | 92 | 90 | 90 |
| Coracoid posterior notch or concavity | 93 | 91 | 91 |
| Intercoracoid facet | 94 | 92 | 92 |
| Humerus anterior flange | 95 | 93 | 95 |
| Plate-like dorsal ridge on humerus | 96 | 94 | 96 |
| Protruding triangular deltopectoral crest on humerus | 97 | 95 | 97 |
| Humerus distal proximal width ratio | 98 | 96 | 98 |
| Humerus with posterodistally deflected ulnar facet and distally facing radial facet | 99 | 97 | 99 |
| Humerus distal articular facets | 100 | 98 | 100 |
| Humerus anterodistal facet for accessory zeugopodial element anterior to radius | 101 | 99 | 101 |
| Humerus/intermedium contact | 102 | 100 | 102 |
| Radius peripheral shaft | 103 | 101 | 105 |
| Radius contiguous shaft | 104 | 102 | 106 |
| Ulna peripheral shaft | 105 | 103 | 109 |
| Ulna contiguous shaft | 106 | 104 | 110 |
| Ulna, olecranon extending posteriorly beyond humerus even if small | 107 | 136 | 112 |
| Shape of the posterior surface of the ulna | 108 | 105 | 111 |
| Radius/ulna relative size | 109 | 106 | 107 |
| Radio-ulnar foramen | 110 | 107 | 108 |
| Radiale, anterior notch | 111 | 108 | 115 |
| Radiale larger than other proximal carpals | 112 | 138 | 114 |
| Manual pisiform | 113 | 109 | 117 |
| Manual pisiform 2 (neomorph) | 114 | 110 | 118 |
| Intermedium | 115 | 111 | 119 |
| Proximal carpals | 116 | 130 | 125 |
| Distal carpal 1 | 117 | 127 | 121 |
| Distal carpal 2 | 118 | 128 | 122 |
| Manual centralia | 119 | 129 | 123 |
| Manual lateral centrale, proximal margin | 120 | 137 | 124 |
| Extra preaxial carpal distally | 121 | 139 | 141 |
| Extra preaxial carpal proximally | 122 | 134 | 140 |
| Mc I peripheral shaft | 123 | 113 | 127 |
| Mc III shaft | 124 | 114 | 128 |
| Mc V | 125 | 115 | 129 |
| Manual digit 2 distal elements peripheral shaft | 126 | 116 | 130 |
| Forelimb hyperphalangy with more than five phalanges ossified in longest digit | 127 | 117 | 133 |
| Manual digit 1 hyperphalangeal and with max number of phalanges | 128 | 142 | 134 |
| Forelimb hypophalangy with less than five digits ossified in longest digit | 129 | 132 | 132 |
| Notching of anterior facet of leading edge elements of forefin in adults, except radiale | 130 | 118 | 116 |
| First preaxial accessory digits on forelimb | 131 | 119 | 138 |
| First phalanx in manual extra anterior digit | 132 | 143 | 142 |
| Second preaxial accessory digit of forelimb | 133 | 126 | 139 |
| Postaxial accessory digit on forelimb | 134 | 120 | 143 |
| Proximal manual phalanges proximo-distal packing | 135 | 121 | 131 |
| Manual digit S4-5 | 136 | 122 | 137 |
| Propodial + epipodial versus manus length | 137 | 123 | 93 |
| Forelimb/hindlimb ratio | 138 | 124 | 94 |
| Forelimb Zeugopodium flattened | 139 | 125 | 103 |
| Delayed mesopodial ossification | 140 | 131 | 126 |
| Carpus elongated, as long as the more distal forelimb part or longer | 141 | 133 | 113 |
| Forelimb zeugopodials shortened relative to humerus | 142 | 135 | 104 |
| Forelimb digits 1-3 'bundled' | 143 | 140 | 135 |
| Forelimb digital separation | 144 | 141 | 136 |
| Iliac blade shape | 145 | 144 | 144 |
| Iliac antero-medial prominence | 146 | 145 | 145 |
| Ilium-pubis relative proximo-distal length | 147 | 153 | 146 |
| Pubis, styloidal or plate-like | 148 | 146 | 147 |
| Pubis obturator foramen | 149 | 147 | 148 |
| Pubis and ischium median symphysis | 150 | 149 | 149 |
| Pubis and ischium fused in adult | 151 | 150 | 150 |
| Pubis ischium relative size | 152 | 151 | 151 |
| Ischium, styloidal or plate-like | 153 | 148 | 152 |
| Thyroid fenestra | 154 | 152 | 153 |
| Femur strongly constricted medially, forming a slender shaft region, proximal width remarkably larger than medial width | 155 | 154 | 154 |
| Femur antero-distal expansion, forming a distinctive structure at the distal end | 156 | 155 | 155 |
| Prominent, ridge-like dorsal and ventral processes demarcated from the head of the femur and extending up to mid-shaft | 157 | 156 | 156 |
| Wide distal femur blade | 158 | 157 | 157 |
| Femur distal facets | 159 | 158 | 158 |
| Femur/astragalus contact | 160 | 159 | 160 |
| Femur anterodistal facet for accessory zeugopodial element anterior to tibia | 161 | 160 | 159 |
| Tibia contiguous shaft | 162 | 161 | 163 |
| Tibia peripheral shaft | 163 | 162 | 164 |
| Tibia antero-proximal end nearly rectangular, forming a deep notch on the anterior margin | 164 | 163 | 165 |
| Fibula posterior extent | 165 | 164 | 166 |
| Fibula contiguous margin | 166 | 165 | 167 |
| Fibula posterior flange | 167 | 166 | 168 |
| Spatium interosseum between tibia and fibula presence | 168 | 168 | 162 |
| Hind fin leading edge element in adults | 169 | 169 | 171 |
| Postaxial accessory digit in hind limb | 170 | 170 | 172 |
| Pes digit 1 | 171 | 171 | 170 |
| Dt 2 in line with dt 3 and 4 | 172 | 172 | 169 |
| Extra anterior metapodial | 173 | 173 | 173 |
| Extra proximal tarsal | 174 | 174 | 174 |
| Atlas/axis fusion | 175 | 175 | 180 |
| Presacral count | 176 | 176 | 175 |
| Cervical count | 177 | 200 | 176 |
| Dorsal count | 178 | 201 | 177 |
| Posterior dorsal centra shape | 179 | 177 | 182 |
| Posterior dorsal/anterior caudal centra degree of shortening | 180 | 178 | 183 |
| Cervical bicipital rib facet | 181 | 179 | 181 |
| Rib articulation in dorsal region | 182 | 180 | 184 |
| Antero-dorsal rib facets | 183 | 181 | 185 |
| Posterior-dorsal bicipital rib facet | 184 | 182 | 186 |
| Sacral ribs | 185 | 183 | 207 |
| Anterior dorsal neural spine | 186 | 184 | 196 |
| Neural spines of atlas-axis | 187 | 185 | 197 |
| Neural spine anticlination in tail | 188 | 186 | 198 |
| Caudal peak with curved vertebral column, near anticlination of neural spine | 189 | 187 | 189 |
| Anterior caudal vertebral size about 1/2 of the largest dorsal vertebrae or less | 190 | 188 | 190 |
| Mid-caudal vertebrae height change | 191 | 189 | 191 |
| Last caudal rib facet reaching the caudal peak area | 192 | 190 | 192 |
| Pre-flexural wedge-shaped centra | 193 | 191 | 193 |
| Preflexual caudal count | 194 | 192 | 178 |
| Tail proportion relative to the rest of the body | 195 | 193 | 179 |
| Lunate tailfin suggested by tailbend | 196 | 194 | 188 |
| Chevrons in caudal-peak region | 197 | 195 | 202 |
| Dorsal neural arch, transverse process | 198 | 196 | 195 |
| Fluke vertebrae, laterally flattened and packed | 199 | 197 | 194 |
| Rib with broadened mid-shaft rostro-caudally | 200 | 198 | 203 |
| Anterior dorsal neural spines with thickened central axis and rostral and caudal median ridges or flanges | 201 | 199 | 199 |
| Anterior dorsal neural spine, second segment | 202 | 202 | 200 |
| Posterior dorsal neural spine, first segment, interspinal space | 203 | 203 | 201 |
| Dorsal rib articulating with two vertebrae | 204 | 204 | 204 |
| Parapophysis elevated above posterior dorsal centra margin | 205 | 205 | 187 |
| Rib posterior flange | 206 | 206 | 205 |
| Anterior dorsal rib, posterior flange extent | 207 | 207 | 206 |
| Ribcage, depth | 208 | 208 | 208 |
| Posterior gastralia immediately craniad of pelvic girdle | 209 | 209 | 209 |
| Flat gastral elements with caudad one outlying the craniad one | 210 | 210 | 210 |
| Dermal ossicle | 211 | 211 | 215 |
| Dermal armor above caudal vertebrae with hemal arches | 212 | 212 | 216 |
| Dermal armor above dorsal neural spines, first layer | 213 | 213 | 217 |
| Lateral gastralia boomerang-shaped, pointing anteriorly | 214 | 214 | 211 |
| Lateral gastral element each approximately symmetrical | 215 | 215 | 212 |
| Median gastralia v-shaped, pointing posteriorly | 216 | 216 | 213 |
| Median gastralia cross-section | 217 | 217 | 214 |
| Dermal armor above dorsal neural spine, third layer | 218 | 218 | 218 |
| Interdigital separation | (removed) | 112 | 120 |
| Spatium interosseum between tibia and fibula size | (removed) | 167 | 161 |
